# Promotion of Lung Cancer Metastasis by SIRT2‐Mediated Extracellular Protein Deacetylation

**DOI:** 10.1002/advs.202205462

**Published:** 2022-12-01

**Authors:** Meng Wu, Jian‐Bin Zhang, Yi‐Wei Xiong, Yong‐Xu Zhao, Meng‐Ge Zheng, Xia‐Li Huang, Fang Huang, Xing‐Xing Wu, Xue Li, Wei‐Jiao Fan, Lin Hu, Yuan‐Yuan Zeng, Xia‐Ju Cheng, Ji‐Cheng Yue, Juan‐Juan Du, Nan‐Nan Chen, Wen‐Xiang Wei, Qing‐Hua Yao, Xiao‐mei Lu, Chao Huang, Jiong Deng, Zhi‐Jie Chang, He‐Bin Liu, Ting C. Zhao, Y. Eugene Chinn

**Affiliations:** ^1^ Institute of Clinical Medicine Research Zhejiang Provincial People's Hospital Hangzhou Medical College Hangzhou Zhejiang 310014 China; ^2^ Institutes of Biological and Medical Sciences Suzhou Medical College of Soochow University Suzhou Jiangsu 215123 China; ^3^ Institute of Health Sciences and Shanghai Key Laboratory for Tumour Microenvironment and Inflammation Shanghai Jiao Tong University School of Medicine Shanghai 200025 China; ^4^ Department of Integrated Chinese and Western Medicine Zhejiang Cancer Hospital Hangzhou Zhejiang 210022 China; ^5^ Clinical Medical Research Institute First Hospital of Xinjiang Medical University Urumqi Xinjiang 830054 China; ^6^ State Key Laboratory of Membrane Biology School of Medicine Tsinghua University Beijing 100084 China; ^7^ Departments of Surgery and Plastic Surgery Rhode Island Hospital Warren Alpert Medical School of Brown University Rhode Island Hospital Providence RI 02903 USA

**Keywords:** acetylation, lung cancer, metastasis, secretion, SIRT2

## Abstract

Acetylation of extracellular proteins has been observed in many independent studies where particular attention has been given to the dynamic change of the microenvironmental protein post‐translational modifications. While extracellular proteins can be acetylated within the cells prior to their micro‐environmental distribution, their deacetylation in a tumor microenvironment remains elusive. Here it is described that multiple acetyl‐vWA domain‐carrying proteins including integrin *β*3 (ITGB3) and collagen 6A (COL6A) are deacetylated by Sirtuin family member SIRT2 in extracellular space. SIRT2 is secreted by macrophages following toll‐like receptor (TLR) family member TLR4 or TLR2 activation. TLR‐activated SIRT2 undergoes autophagosome translocation. TNF receptor associated factor 6 (TRAF6)‐mediated autophagy flux in response to TLR2/4 activation can then pump SIRT2 into the microenvironment to function as extracellular SIRT2 (eSIRT2). In the extracellular space, eSIRT2 deacetylates ITGB3 on aK416 involved in cell attachment and migration, leading to a promotion of cancer cell metastasis. In lung cancer patients, significantly increased serum eSIRT2 level correlates with dramatically decreased ITGB3‐K416 acetylation in cancer cells. Thus, the extracellular space is a subcellular organelle‐like arena where eSIRT2 promotes cancer cell metastasis via catalyzing extracellular protein deacetylation.

## Graphical abstract

SIRT2 is secreted into the microenvironment from macrophages upon stimulation. SIRT2 is detected within the autophagosome and the autophagic apparatus serves as conduits for the transport of SIRT2 across plasma membrane. Intriguingly, SIRT2 promotes cancer cell metastasis presumably via deacetylating multiple extracellular proteins including ITGB3 and collagens. It provide new means by targeting SIRT2 in the microenvironment in order to block metastasis.

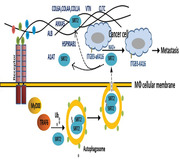

## 1 Introduction

Considerable efforts have been made to elucidate the biological function of how the extracellular microenvironment affects cancer cell metastasis.^[^ ^1^, ^2^ ^]^ Proteins in the extracellular space include extracellular matrix (ECM) proteins, cytokines, enzymes, and cellular transmembrane receptors. A significant portion of extracellular and pericellular proteins are phosphorylated and acetylated.^[^ ^3^, ^4^ ^]^ Acetylation of a wide range of ECM and pericellular proteins has been reported by high‐throughput liquid chromatography tandem mass spectrometry analysis in both healthy and diseased tissues, where the levels of acetylation are dynamically regulated.^[^ ^3^, ^5^, ^6^ ^]^ Accumulating evidence has revealed the existence of extracellular localized kinase and phosphatase, suggesting the importance of dynamic extracellular protein phosphorylation.^[^ ^4^, ^7^, ^8^, ^9^, ^10^ ^]^ However, the significance of extracellular protein acetylation is still not fully recognized due to the lack of known extracellular enzymes.

In many of those extracellular proteins, acetylated lysine residues were found in functionally conserved domains in distantly related proteins or conserved motifs among different species,^[^ ^6^ ^]^ suggesting a conserved regulatory role for these modifications. For example, acetylated lysine residues were found at equivalent vWA domains in collagens and integrins.^[^ ^5^ ^]^ Remarkably, acetylation at Lys292 in mouse*α*1‐antitrypsin (A1AT, the major extracellular serine proteases inhibitor), was previously shown to be dramatically elevated upon conventionalization of gnotobiotic mice. The orthologous residue in human A1AT was also acetylated.^[^ ^6^ ^]^ These extracellular proteins, such as High mobility group box1 (HMGB1) and Heat shock protein90 (HSP90), two abundant proteins in the ECM, can be acetylated by the Golgi/ER localized acetyltransferases prior to their secretion.^[^ ^11^ ^]^ To reverse the acetylation, the secretion of deacetylases into the extracellular space is necessary.^[^ ^6^ ^]^

NAD^+^‐dependent Sirtuin family member SIRT2 is a cytoplasmic enzyme involved in the regulation of microtubule dynamics by deacetylating *α*‐tubulin.^[^ ^12^ ^]^ SIRT2 also has been reported to deacetylate p53, nuclear factor kappa B (NF‐κB), APC, SMC1A, and a number of metabolic enzymes.^[^ ^13^, ^14^ ^]^ An observation from a proteomic analysis of mouse macrophages demonstrated that SIRT2 was detected in the culture medium together with 775 proteins.^[^ ^15^ ^]^ SIRT2 was subsequently reported for its presence in human serum and was thought to participate in age‐related diseases including atypical Parkinson's syndromes.^[^ ^16^, ^17^ ^]^ These results implied that SIRT2 might be the enzyme responsible for extracellular protein deacetylation.

Macrophages are a type of cell involved with cytokine secretion. Most cytokines depend on their N‐terminal signal peptide (SP), a hydrophobic 20–30 residues span, to bypass the hydrophobic lipid membrane for secretion. However, macrophages also secrete those proteins without typical signaling peptides such as interleukin (IL)‐1*β*, IL‐18, and HMGB1 via secretory autophagy.^[^ ^18^, ^19^ ^]^ In the cancer microenvironment, about half of infiltrating cells are macrophages and depletion of these cells inhibited tumor growth and metastasis.^[^ ^20^ ^]^ Tumor associated macrophages (TAMs) secrete cytokines or growth factors to the sites where most inflammatory cells, including macrophages co‐reside and determine the proliferation and metastasis of a tumor.^[^ ^21^, ^22^ ^]^ The secretion characteristics of TAMs are largely dependent on the stimuli present in the tumor microenvironment.^[^ ^23^ ^]^ Based on their stimuli and functions, macrophages can be classified into M1 and M2 categories. M2 macrophages have pro‐tumor features similar to TAMs, whereas M1 macrophages exhibit anti‐tumor properties. However, this classification is somewhat over‐simplified since M1 and M2 macrophages often express genes cross the line between M1 and M2 macrophages.^[^ ^24^, ^25^ ^]^ Although 70% of TAMs are of the M2 subtype in non‐small‐cell lung cancer (NSCLC), a higher density of M1 macrophages was found in metastatic NSCLC patients versus non‐metastatic patients.^[^ ^26^ ^]^

The RING‐domain E3 ligase TRAF6 activation in response to TLRs as well as other transmembrane receptors triggers signaling pathways leading to NF‐kB activation, autophagosome flux, and cytokine secretion.^[^ ^27^, ^28^ ^]^ Autophagy triggers protein secretion independent of these well‐known protein secretory signaling pathways.^[^ ^29^, ^30^ ^]^ We report here that SIRT2 was secreted into the microenvironment from macrophages upon stimulation. SIRT2 was detected within the autophagosome in macrophages treated with TLR4/TLR2 activating ligands. Moreover, SIRT2 secretion was significantly decreased in autophagy related 7 (ATG7)‐depleted macrophages. Hence, the autophagic apparatus served as conduits for the transport of SIRT2 across plasma membrane. Intriguingly, SIRT2 promoted cancer cell metastasis presumably via deacetylating multiple extracellular proteins including ITGB3 and collagens in the microenvironment. Elevated levels of SIRT2 in the serum, along with decreased ITGB3‐K416 acetylation in lung cancer patients provided new means by targeting SIRT2 in the microenvironment in order to block metastasis.

## 2 Results

### 2.1 SIRT2 Is Secreted by Macrophages upon TLR Activation

**Figure 1:**
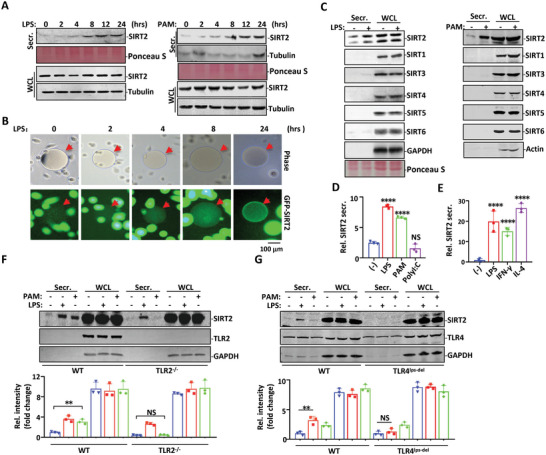
SIRT2 secrets from macrophages upon TLR4 or TLR2 activation. A) Mouse peritoneal macrophages were treated with LPS or PAM for indicated times. Proteins in cell culture supernatants (Secr.) were concentrated with trichloroacetic acid (TCA) precipitation and stained with Ponceau S while cells were lysed with RIPA buffer as WCL. Both Secr. and WCL fractions were analyzed in Western blot with SIRT2 antibody. A weak Tubulin secretion was detected with PAM treatment (right panel). B) THP1 cells stably expressing GFP‐SIRT2 were primed with phorbol 12‐myristate 13‐acetate (PMA) for 2 days followed by LPS treatment for indicated times. SIRT2 antibody conjugated agarose beads (red arrows) were added into the cell culture and the GFP‐SIRT2 signal trapped by the beads was then visualized with a fluorescent microscope. C) THP1 cells primed with PMA were treated with LPS or PAM for 24 h. TCA precipitated cell supernatant (Secr.) and WCL were analyzed in Western blot using antibodies against SIRT family members as indicated. D,E) Mouse peritoneal macrophages were treated with different ligands as indicated for 24 h in (D) and (E). SIRT2 abundance in cell supernatant (Secr.) and WCL samples were analyzed in Western blot with SIRT2 antibody or GAPDH/β‐actin antibody. Densitometric analysis was performed using ImageJ software and plotted in the histograms. Peritoneal macrophages collected from wild‐type, F) TLR2^−/‐^ mice or G) TLR4^LPS‐del^ mice were treated with LPS or PAM. Cell culture medium (Secr.) and WCL were then analyzed in Western blot for SIRT2 secretion. Densitometric analysis was conducted using ImageJ software. Data are shown as mean ± SEM. **, *p* < 0.01, ****, *p* < 0.0001; NS indicates no significant difference between mock‐treated (‐) and the indicated treatment group or between indicated two groups.

While investigating the properties of macrophages in modulating extracellular protein acetylation, SIRT2 emerged as a promising candidate as a secreted deacetylase.^[^ ^15^ ^]^ SIRT2 proteins were accumulated in a time dependent manner, as found in the medium collected from mouse peritoneal macrophages treated with lipopolysaccharide (LPS) (**Figure** **1**A). SIRT2 secretion from macrophages was also dramatically induced by TLR2 ligand Pam_3_CSK_4_ (PAM) (Figure 1A) or peptidoglycan (PGN) (Figure S1A, Supporting Information). Both TLR4 and TLR2 ligands were equally effective in SIRT2 secretion induction (Figure S1B, Supporting Information). Although macrophages are highly heterogeneous depending on their origins, a similar hyper‐secretary pattern of SIRT2 was observed in macrophage cell lines from different origins or species (Figure S1C, Supporting Information).

In order to validate SIRT2 secretion, we performed the bead halo assay, which has been previously used to determine protein‐protein interactions in cells.^[^ ^31^, ^32^ ^]^ THP1 cells stably expressing GFP‐SIRT2 (THP1‐GFP‐SIRT2) were differentiated by PMA and treated with LPS. Secreted GFP‐SIRT2 proteins were trapped by the beads conjugated with SIRT2 antibody added into the medium (Figure 1B). In response to prolonged LPS stimulation, the GFP‐fluorescent signal increased gradually over time (Figure 1B). Among all sirtuin family members, SIRT2 was the only one secreted in response to LPS or PAM treatment (Figure 1C). The fact that both LPS and PAM induced TNF‐*α* gene but not SIRT2 gene for expression (Figure S1D, Supporting Information) suggests that gene expression and protein secretion are two distinct signaling events in response to TLR4 or TLR2 activation.

Activated macrophages can be broadly categorized into M1 and M2 subsets. M1 polarization is triggered by pro‐inflammatory TLR‐agonists or cytokines, such as LPS and interferon (IFN)*γ*. M2 status is activated by cytokines IL‐4 and IL‐13.^[^ ^22^ ^]^ Unlike TLR4 activation by LPS, which markedly induced M1 polarization, activation of TLR2 by PAM or TLR3 by PolyI:C had less of an effect on M1 polarization (Figure S1E, Supporting Information). Intriguingly, while both LPS and PAM were effective in inducing SIRT2 secretion, PolyI:C failed to induce secretion in SIRT2 or other SIRT family members (Figure 1D and Figure S1F,G, Supporting Information). The fact that both IFN‐*γ* and IL‐4 induced SIRT2 secretion (Figure 1E and Figure S1H, Supporting Information) suggests SIRT2 secretion is independent of macrophage polarization.

The role of TLR4 and TLR2 in SIRT2 secretion was further confirmed in TLR4^−LPS‐del^ and TLR2^−/−^ mice respectively. As expected, in peritoneal macrophages collected from TLR4^−LPS‐del^ and TLR2^−/−^ mice, TLR4 ligand LPS and TLR2 ligand PAM failed to induce SIRT2 secretion respectively (Figure 1F,G). Thus, both TLR2 and TLR4 are involved in SIRT2 protein secretion induction in macrophages.

### 2.2 TRAF6 E3 Ligase Pathway Is Critical for SIRT2 Secretion

**Figure 2:**
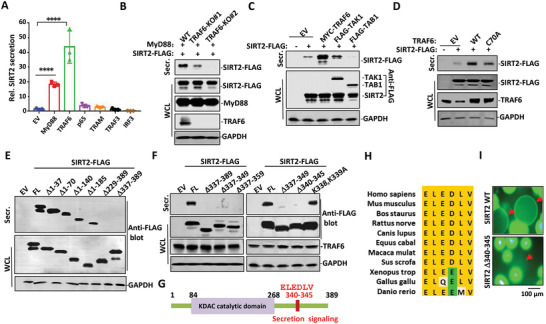
TRAF6 mediates SIRT2 secretion in activated macrophages. A) SIRT2 was transfected with empty vector (EV), MyD88, TRAF6, p65, TRAM, or TRAF3 in HEK293T cells. The cell culture supernatants were collected for SIRT2 protein measurement using ELISA. Data are shown as mean ± SEM. ****, *p* < 0.0001 between EV (empty vector) and MyD88 group or between EV group and TRAF6 group. B) Wild‐type or TRAF6‐KO HEK293T cells were transfected with MyD88 and SIRT2‐FLAG. Secreted SIRT2‐FLAG was enriched with anti‐FLAG M2 agarose beads and eluted with sample buffer. WCL and eluate were subjected to Western blot analysis using antibodies against FLAG, MyD88, or TRAF6. C) SIRT2‐FLAG was co‐transfected with EV, TRAF6, TAK1, or TAB1 in HEK293T cells. SIRT2‐FLAG secreted into culture medium (Secr.) was enriched with anti‐FLAG M2 agarose beads and subjected, together with WCL, to Western blot analysis using FLAG antibody. D) SIRT2‐FLAG was co‐transfected with wild‐type TRAF6 or catalytically inactive TRAF6 (C70A) mutant in HEK293T cells. Secreted SIRT2‐FLAG and the expressions of transfectants were analyzed in Western blot. E) Various N‐terminal or C‐terminal domain deletion mutants of SIRT2 in FLAG tagged form were transiently expressed in HEK293T cells. Secreted SIRT2‐FLAG proteins (enriched with M2 beads) were analyzed by Western blot with FLAG antibody. F) A series of C‐terminal domain deletion variants, as well as K338A/K339A mutant of SIRT2 in FLAG tagged form were constructed and transiently expressed in HEK293T cells. Secreted SIRT2‐FLAG proteins (enriched with M2 beads) were analyzed in Western blot with FLAG antibody. G) Schematic drawing of human SIRT2 shows the secretion signaling motif. H) Sequence alignment of SIRT2 secretion signaling motif across different species. I) GFP‐SIRT2 WT or GFP‐SIRT2 Δ340–345 expressing THP1 cells were differentiated with PMA followed by LPS treatment in the presence of SIRT2 antibody conjugated agarose beads (red arrows). GFP (SIRT2) halo on the beads surface was visualized with fluorescent microscope.

The canonical TLR signaling events include both myeloid differentiation factor 88 (MyD88)‐dependent and MyD88‐independent pathways. We examined the signaling adaptors and transcription factors involved in these two pathways for SIRT2 secretion. Overexpression of either MyD88 or TRAF6 induced a substantial increase in SIRT2 secretion in HEK293T cells as determined by enzyme linked immunosorbent assay (ELISA) and Western blot analysis (**Figure** **2**A and Figure S2A, Supporting Information). In contrast, overexpression of TRIF‐related adaptor molecule (TRAM) and TRAF3, two crucial components of the MyD88‐independent pathway, failed to induce SIRT2 secretion (Figure 2A and Figure S2B, Supporting Information). However, in TRAF6 depleted HEK293T cells, MyD88 by itself failed to induce SIRT2 secretion (Figure 2B). This suggests that MyD88 relies on TRAF6 signaling for SIRT2 secretion. MyD88 is composed of an N‐terminal Death domain (DD) and C‐terminal Toll/Interleukin‐1 receptor (TIR) domain. Interestingly, the secretion of SIRT2 was contingent on the N‐terminal DD of MyD88, while the C‐terminal TIR domain almost completely abolished SIRT2 secretion (Figure S2C, Supporting Information). This is reasonable given that the DD of MyD88 is responsible for the assembly of heterotrimeric Myddosome, which further interacts with TRAF6 to facilitate downstream activation.^[^ ^33^ ^]^ The TIR domain mediates the interactions with TLR2/TLR4 receptor and acts as a dominant negative when expressed alone.^[^ ^27^, ^34^ ^]^ The positive effect of TRAF6, on SIRT2 secretion was confirmed by co‐transfection of SIRT2 with TRAF6 in HEK293T cells (Figure 2C). Transforming growth factor‐*β* (TGF‐*β*)‐activated kinase 1 (TAK1) through assembling with its binding partner TAK1‐binding protein 1 (TAB1) can serve as the immediate downstream signaling factors of TRAF6 leading to the activation of transcription factor NF‐κB. Ectopic expression of TAK1, TAB1 or NF‐κB (p65) failed to affect SIRT2 secretion in HEK293T cells (Figure 2A,C). Together, these results demonstrate that TRAF6 is the checkpoint for SIRT2 secretion, which does not rely on NF‐κB activation.

We next investigated the intrinsic E3 ligase activity of TRAF6 in SIRT2 secretion. While overexpression of wild‐type TRAF6 strongly enhanced SIRT2 secretion, overexpression of TRAF6‐C70A mutant, an E3 catalytic inactive form, largely abolished its activity inSIRT2 secretion (Figure 2D). This indicates that the intrinsic E3 ligase activity of TRAF6 was involved in SIRT2 secretion stimulation. As expected, overexpression of wild‐type TRAF6 but not the TRAF6‐C70A mutant induced SIRT2 ubiquitination in HEK293T cells (Figure S2D, Supporting Information). To corroborate this result, we reconstituted an in vitro E1‐E2‐TRAF6 ubiquitination system using purified proteins by using His‐SIRT2 as the substrate. The linear ubiquitin chains were generated in this in vitro system, indicating that SIRT2 is a bona fide substrate of TRAF6 (Figure S2E, Supporting Information).

Many typical secretory proteins bear an N‐terminal signaling peptide. When N‐terminal residues were gradually deleted from 37 up to 185, SIRT2 was still secreted in HEK293T cells (Figure 2E). In contrast, a 52 amino acid‐deletion on the C‐terminus spanning residues 337–389 largely abolished SIRT2 secretion (Figure 2F and Figure S2F, Supporting Information). Additional efforts allowed us to narrow down the minimal motif responsible for SIRT2 secretion to residues 340–345 (ELEDLV) (Figure 2F,G and Figure S2F, Supporting Information), which is conserved among a wide range of species (Figure 2H). Mutations of K338, K339, or L341 in close proximity to this motif did not affect SIRT2 secretion (Figure 2F and Figure S2G, Supporting Information). To further validate that the 340–345 motif is required for SIRT2 secretion, THP1 cells stably expressing wild type or Δ340–345 SIRT2 in GFP form were primed with PMA followed by LPS stimulation in the presence of SIRT2 antibody‐conjugated beads. GFP‐fluorescent‐beads were only visualized in the culture medium of THP1 cells expressing GFP‐SIRT2 in wild type form but not in the culture medium of THP1 cells expressing GFP‐SIRT2 with Δ340–345 form (Figure 2I and Figure S2H, Supporting Information). We, therefore, defined this “ELEDLV” sequence as the specific secretion motif of the SIRT2 protein.

### 2.3 Autophagosome Forms a SIRT2 Secretion Flux

**Figure 3:**
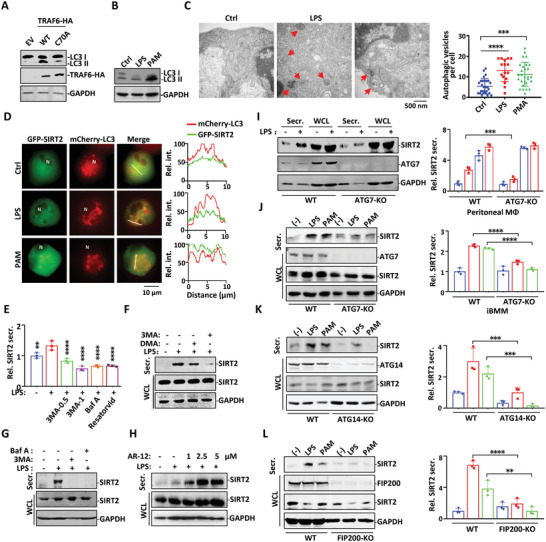
The autophagic machinery is utilized for SIRT2 secretion. A) HEK293T cells transfected with either wild type or catalytic‐defective C70A mutant of TRAF6 were subjected to Western Blot analysis for LC3 expression. B) Mouse peritoneal macrophages were treated with LPS or PAM for 24 h, followed by Western blot analysis with antibodies against LC3 and GAPDH. C) Transmission electron microscopy of mouse peritoneal macrophages treated with LPS or PAM for 24 h. Arrows indicate autophagic vesicles. The number of autophagic vesicles per cell was plotted. D) THP1 cells transfected with GFP‐SIRT2 and mCherry‐LC3 were primed with PMA for 48 h, followed by stimulation with LPS or PAM for 24 h. The intracellular distributions of SIRT2 and LC3 were visualized by fluorescent microscopy. Intensity profiles were generated from the white lines using ImageJ. E) Mouse peritoneal macrophages were pre‐treated with DMSO, 3MA (0.5, 1 mmm), Baf A (50 nm), or Resatorvid (50 µm) for 1 h followed by LPS treatment. Cell culture supernatants were collected and subjected to SIRT2 ELISA analysis. Statistics difference was compared to the LPS treatment group. F) Mouse peritoneal macrophages were pre‐treated with DMSO, 1 mm 3MA, or 100 nm DMA for 1 h followed by LPS treatment. SIRT2 proteins of Secr and WCL fractions were analyzed in Western blot. G) Mouse peritoneal macrophages were pre‐treated with 3MA or Baf A followed by LPS treatment. SIRT2 proteins of Secr and WCL fractions were analyzed in Western blot. H) PMA primed THP1 cells were treated with increasing concentrations of AR‐12 for 1 h, followed by LPS stimulation for 24 h. SIRT2 of Secr. and WCL fractions were analyzed in Western blot. I) SIRT2 secretion was induced by LPS treatment in peritoneal macrophages obtained from wild type and ATG7‐KO mice. Densitometric readings were normalized to those in the resting WT group and plotted in the bar graphs. J) ATG7‐KO, K) ATG14‐KO, L) Focal adhesion kinase family interacting protein of 200 kD (FIP200)‐KO or wild type immortalized bone marrow‐derived macrophages (iBMMs) were treated with LPS or PAM for 24 h. SIRT2 proteins of Secr. and WCL fractions were analyzed in Western blot. Densitometric readings were normalized to those in the resting WT group and plotted in the bar graphs. Data are shown as mean ± SEM. **,*p* < 0.01; ***,*p* < 0.001; ****, *p* < 0.0001 between the indicated treatment groups.

Given that TRAF6 overexpression converts microtubule‐associated protein 1A/1B‐light chain 3 (LC3) from cytoplasmic form LC3‐I to lipid‐bound form LC3‐II in autophagosome formation,^[^ ^35^ ^]^ we wondered whether secretory autophagy is the underlying cause of SIRT2 secretion. Treatment with LPS or PAM or ectopic overexpression of TRAF6 could all induce the conversion of LC3‐I to LC3‐II involved in the formation of bilayer autophagic vesicles (**Figure** **3**A,B).^[^ ^35^ ^]^ Transmission electron microscopy confirmed the formation of bilayer autophagic vesicles in LPS‐ or PAM‐ treated macrophages (Figure 3C and Figure S3A, Supporting Information). A substantial amount of cytoplasmic SIRT2 colocalized with LC3 in response to LPS and PAM treatment (Figure 3D). Hence, SIRT2 could be a potential substrate of autophagy machinery. The autophagy inhibitor 3‐Methyladenine (3MA) and bafilomycin A1 (Baf A) exhibited a significant inhibition of SIRT2 secretion as compared to the exosome inhibitor 5‐(*N*,*N*‐dimethyl)‐amiloride hydrochloride (DMA) (Figure 3E–H). Although glycosylation is known to be an essential component for secretion of many cellular proteins,^[^ ^36^ ^]^ *N*‐glycosylation inhibitor Tunicamycin failed to affect SIRT2 secretion (Figure S3B, Supporting Information), suggesting that glycosylation does not play any role in SIRT2 secretion. Rather, the autophagy activator AR‐12 markedly stimulated SIRT2 secretion (Figure 3H). These findings suggest that TLR2 or TLR4 activation‐induced autophagy flux was able to pump SIRT2 proteins out of macrophages.

It is well known that loss of ATG7 impairs the formation of the LC3‐autophagosome vesicle body, resulting in a deficiency or blocking effect of the onset of autophagy‐related activities in cells.^[^ ^37^ ^]^ In the macrophages obtained from myeloid cell specific ATG7 deficient mice (LysMCre; ATG7^flox/flox^, hereafter referred as ATG7‐KO), LPS‐induce SIRT2 secretion was significantly impaired (Figure 3I). Furthermore, we evaluated various components of the autophagy pathway in SIRT2 secretion by generating stable gene knockout macrophages using CRISPR‐Cas9 technology. Depletion of ATG7, ATG14, or FIP200 all markedly impaired SIRT2 secretion in response to LPS and PAM stimulation (Figure 3J–L). Thus, autophagosome flux formation is the driving force for efficient SIRT2 protein secretion in macrophages in response to TLR4 or TLR2 activation.

### 2.4 SIRT2 Deacetylates Multiple Extracellular Proteins

**Figure 4:**
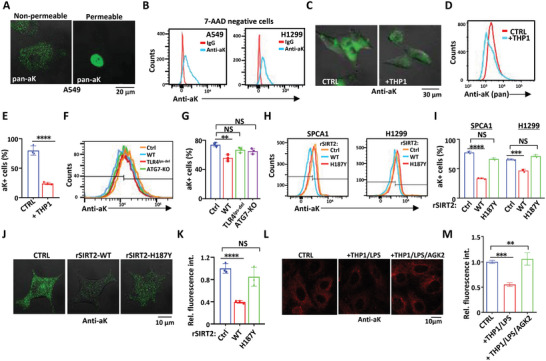
Secreted SIRT2 deacetylates membrane proteins extracellularly in lung cancer cells. A) Viable (left) or formaldehyde fixed/Triton X‐100 permeabilized (right) A549 cells were immuno‐stained with pan anti‐acetyl‐K antibody followed by Alexa Fluor 488 conjugated secondary antibody. Cells were visualized with confocal fluorescent microscope. B) A549 and H1299 cells were stained with 7‐AAD and pan acetyl‐K antibody followed by flow cytometry analysis. Cells stained negative for 7‐AAD staining (with intact membranes) were analyzed for acetylation intensity. C–E) A549 cells were co‐cultured with or without LPS‐stimulated THP1 cells. Cells were then non‐permeably stained with pan acetyl‐K antibody and Alexa Fluor 488 conjugated secondary antibody followed by visualization under C) confocal microscopy or D,E) analysis with flow cytometry. F,G) A549 cells were left untreated (Ctrl) or treated with conditioned medium harvested from LPS stimulated peritoneal macrophages from WT, TLR4^LPS‐del^, or ATG7‐KO mice. Cells were then stained with pan acetyl‐K antibody and Alexa Fluor 488 conjugated secondary antibody, followed by flow cytometry analysis. H,I) SPCA1 and H1299 cells were either untreated or treated with rSIRT2 WT or rSIRT2‐H187Y proteins. Cells were then stained with pan acetyl‐K antibody and Alexa Fluor 488 conjugated secondary antibody, followed by flow cytometry analysis. J) Extracellular membrane acetylation of A549 cells received mock, rSIRT2 WT, or rSIRT2‐H187Y treatment were visualized with confocal microscope using non‐permeable pan acetyl‐K antibody for staining. K) Median fluorescence intensities (MFIs) of these A549 cells were quantified. L) A549 cells were co‐cultured with LPS‐stimulated THP1 cells in the presence or absence of SIRT2 inhibitor AGK2. Cells were then non‐permeably stained with pan acetyl‐K antibody and Alexa Fluor 594 conjugated secondary antibody followed by visualization under confocal microscopy. M) Intensity profiles were generated in different groups using ImageJ. Data are shown as mean ± SEM. **, *p* < 0.01; ***, *p* < 0.001; ****, *p* < 0.0001; NS suggests no significant difference between CTRL and the indicated group.

The Acetylome profile revealed that proteins in ECM or cell membrane are extensively acetylated.^[^ ^5^, ^6^, ^38^ ^]^ A punctate pattern of acetylation signal was visualized on the surface of A549 lung cancer cells by using a pan‐acetylation antibody for cell membrane non‐permeable immuno‐staining (**Figure** **4**A). In contrast, nuclear acetyl‐proteins of A549 cells were highly condensed using the same antibody for permeable staining (Figure 4A). Similarly, more than 80% of the membrane intact (7‐AAD‐negative) lung cancer cells stained positive for lysine acetylation as assessed by flow cytometric analysis (Figure 4B). These results suggest that the extracellular domains of cellular trans‐membrane proteins were strongly acetylated in these lung cancer cells.

Next, the deacetylation effect of SIRT2 secretion from macrophages on cancer cell membrane protein was evaluated by co‐culturing THP1 cells with A549 cells in a transwell system where the two types of cells were separated by 0.4 µm pore membranes. The number and intensity of acetylation‐positive A549 cells were much fewer or weaker than that of A549 cells incubated alone (Figure 4C–E). Likewise, conditioned medium from LPS stimulated peritoneal macrophages of wild‐type mice decreased the acetylation level on A549 cell surface. In contrast, conditioned medium collected from both TLR4‐LPS‐del and ATG7‐KO macrophages failed to do so (Figure 4F,G). We then incubated lung cancer cell lines SPCA1, H1299, and A549 with recombinant SIRT2 protein (rSIRT2) purified from bacteria. Addition of wild type rSIRT2 but not the catalytic inactive rSIRT2 (rSRIT2‐H187Y) proteins decreased the frequency of cells that stained positive for extracellular membrane‐acetylation as well as the acetylation intensity in these lung cancer cell lines (Figure 4H–K). In the presence of LPS‐activated THP1 cells, the extracellular protein acetylation intensity of A549 lung cancer cells was significantly reduced (Figure 4L,M). When AGK2, a lipid‐membrane associable SIRT2 inhibitor, was included in the system, the A549 cell membrane acetylation intensity was restored (Figure 4L,M). These results clearly revealed a deacetylation catalytic activity of secreted SIRT2 proteins on pericellular proteins.

**Figure 5:**
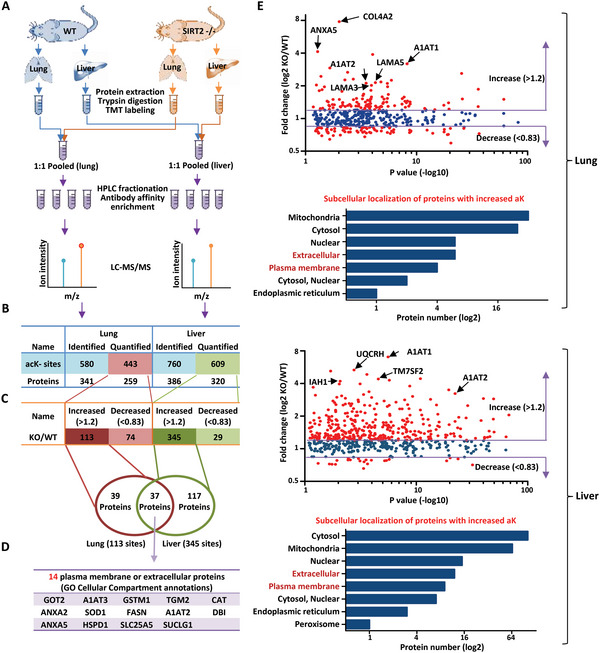
Identification of extracellular deacetylation proteomics in SIRT2^−/‐^ mice. A) Workflow for Isobaric TMT based acetylome analysis of mice lung and liver tissues. Integrated approach involving TMT labeling, HPLC fractionation, affinity enrichment, mass spectrometry‐based quantitative proteomics was used to quantify dynamic changes of lysine acetylome between SIRT2^−/‐^ mice and their wild type littermates. B) Quantitative overview of each TMT experimental database search of lung and liver organs. C) In lung tissues, 580 acetyl‐K sites in 341 protein groups were identified, among which 443 sites in 259 proteins were quantified and 113 sites were up‐regulated, 74 sites were down‐regulated in SIRT2^−/‐^ mice when compared to their wild‐type littermates. In liver tissues, 760 acetyl‐K sites in 386 protein groups were identified from mouse *liver*, among which 609 sites in 320 proteins were quantified and 345 sites were up‐regulated, 29 sites were down‐regulated. D) Venn diagram of proteins with significantly up‐regulated lysine acetylation (SIRT2^−/‐^ versus WT) from each experimental group comparison. Membrane and extracellular proteins in the overlapping region are presented below. E) Scatter plots depicting the log2(fold change) of lysine acetylation(normalized to protein abundance) versus ‐log10 (*p*‐value) for acetyl‐peptides. Proteins harbor significantly up‐regulated (fold change > 1.2) or down‐regulated (fold change < 0.83) acetyl‐K sites in SIRT2^−/‐^ lung (upper) or liver (below) tissues are in red. Example membrane or extracellular proteins bearing dramatically increased acetyl‐Ks are highlighted. Collagen IVa2 (COL4A2); Annexin A5 (ANXA5); Serpin Family A Member 1 (A1AT1 and A1AT2); Laminina5 (LAMA5); Laminina3 (LAMA3); Ubiquinol‐Cytochrome C Reductase Hinge Protein (UQCRH); Transmembrane 7 Super‐family Member 2 (TM7SF2); Isoamyl Acetate‐Hydrolyzing Esterase 1 Homolog (IAH1). Histograms depict subcellular localization of those proteins with upregulated acetyl‐Ks according to Gene Ontology (GO) annotations. The number of proteins in each cluster is indicated beside the bars.

To further quantify the extracellular protein substrates of SIRT2, proteins of lung and liver tissues from 8‐week‐old wild type and SIRT2^−/−^ mice were subjected to tandem mass tag (TMT*)* mass spectrometry for acetylome analysis (**Figure** **5**A). Protein samples were labeled with sets of isobaric compounds on primary amine groups. Each isobaric reagent contains a different number of heavy isotopes in the mass reporter region, resulting in a unique reporter mass for protein modification motif identification and quantitation. Three hundred and forty‐one proteins with 580 acetylation sites from mouse lung and 386 proteins with 760 acetylation sites from mouse liver were identified respectively (Figure 5B). More importantly, 113 peptides carrying acetyl‐K residues were found upregulated, and 74 peptides downregulated in SIRT2^−/−^ lung tissues as compared to the wild type, when applied a cut‐off in peptide abundance as 1.2 (Log_2_(fold‐change)) (Figure 5C). Compared with the lung, the liver had approximately three fold more peptides with acetylation intensity up‐regulated (i.e., 345 versus 113) whereas close to three fold fewer (i.e., 29 versus 79) peptides with acetylation intensity down‐regulated (Figure 5C). The Venn diagram revealed that within the up‐regulated peptides, 37 corresponding proteins were commonly identified in both lung and liver tissues, 14 of which were localized in the plasma membrane or were extracellular according to their GO cellular compartment annotations (Figure 5D). These findings indicate SIRT2 targets a broad range of proteins in particular the plasma membrane or extracellular proteins for deacetylation in both liver and lung tissues.

Quite a few extracellular proteins were upregulated in their acetylation intensity in SIRT2^−/−^ lung and liver as their enrichment in cellular compartment GO category (Figure 5E). Compared with intracellular (i.e., cytosolic, nuclear, and mitochondrial) proteins, extracellular proteins displayed a more robust increase in acetylation intensity from both lung and liver of SIRT2^−/−^ mice (Figure 5E). The acetyl‐lysine was found in the conserved vWA domain of many proteins, for example, integrins and Collagen‐type Vi*α* (COL6A1and COL6A2) (Figure 5E and Tables S1 and S2, Supporting Information). vWA domain is quite conserved in many extracellular proteins involved in cell micro‐environmental activity regulation.

**Figure 6:**
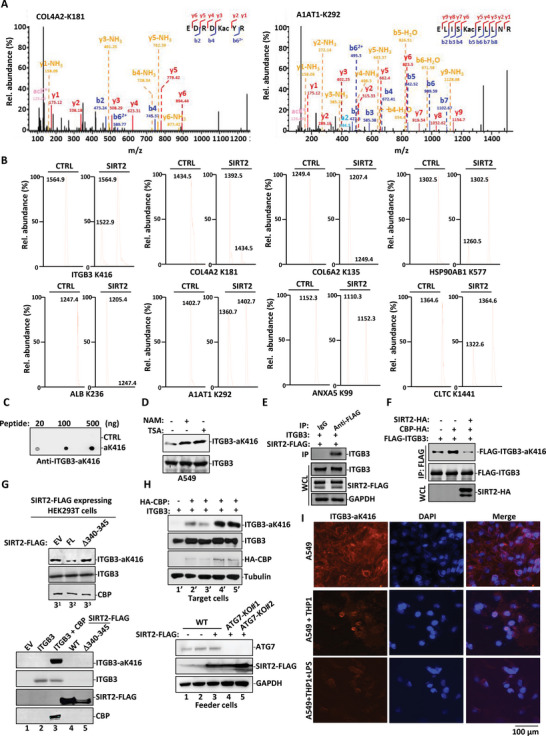
SIRT2 deacetylates the vWA domain of ITGB3. A) MS/MS spectra of 2 peptides with up‐regulated K acetylation, including Col4a2 K181 (EDRDK(ac)YR) and Serpina1a K292 (ELISK(ac)FLLNR) were recovered from SIRT2^−/‐^ mouse lung tissues. B) Synthetic peptides with indicated K acetylation were incubated with or without rSIRT2 protein tagged with His in the presence of NAD+ followed by linear mode MALDI‐TOF‐MS analysis. The mass reduction of 42 daltons in mass spectrum confirmed the SIRT2‐catalyzed lysine deacetylation reaction. C) Polyclonal antibody against acetyl‐K416 within the vWA domain of ITGB3 (using synthesized acetyl‐peptide of K416 motif as the antigen) was generated in rabbit and validated by dot blot experiment. This antibody was reactive with ITGB3‐aK416 peptide in a concentration dependent manner while it had no immuno‐reactivity toward the unacetylated control peptide. D) A549 cells were treated with NAM or TSA. WCL was blotted with the ITGB3‐aK416 antibody and ITGB3 antibody. E) HEK293T cells were transfected with the indicated plasmids. SIRT2‐FLAG was enriched with anti‐FLAG M2 agarose beads and the expression level of ITGB3 was analyzed using Western blotting. F) HEK293T cells were transfected with the indicated plasmids. ITGB3‐Flag was enriched with anti‐FLAG M2 agarose beads and K416 acetylation was analyzed with either ITGB3‐aK416 antibody or pan acetyl‐K antibody. G) HEK293T cells were transfected with indicated plasmids (i.e., EV, ITGB3, ITGB3 plus CREB‐binding protein (CBP), SIRT2 WT, SIRT2 Δ340–345) in lower panel. An aliquot of cells transfected with ITGB3 plus CBP was co‐cultured for 36 h with 1: EV transfected cells, 2: SIRT2 WT transfected cells, 3: SIRT2 Δ340–345 transfected cells (upper panel). Cells were then lysed in 2× SDS sample buffer and subjected to Western blot analysis with indicated antibodies. H) ITGB3‐aK416 acetylation was evaluated in HEK293T cells transiently expressing ITGB3 along with or without HA‐CBP (target cells, upper panel) and cultured with supernatant collected from WT or ATG7KO HEK293T cells transfected with EV or SIRT2‐FLAG (feeder cells, lower panel). WCL were extracted and subjected to Western blot analysis using antibody as indicated. I) A549 cells were cultured alone or co‐cultured with LPS‐treated or untreated THP1 cells. THP1 cells were removed with PBS wash and A549 cells were then non‐permeably stained with anti‐ITGB‐aK416 antibody and Alexa Fluor 594 conjugated secondary antibody followed by visualization under confocal microscopy.

COL4A2 acetylation on K181 (peptide “EDRDK(ac)YR”) displayed a seven fold elevation in acetylation intensity in SIRT2^−/−^ mouse lungs (**Figure** **6**A and Table S1, Supporting Information). A1AT acetylation on K292 (peptide “ELISK(ac)FLLNR”) displayed more than three fold and seven fold elevation in SIRT2^−/−^ mouse lungs and livers, respectively (Figure 6A and Tables S1 and S2, Supporting Information). A1AT K292 acetylation was also identified as highly elevated in colon during the conventionalization of gnotobiotic mice.^[^ ^6^ ^]^ We then performed in vitro analysis to confirm deacetylation of these protein motifs by SIRT2. Acetyl‐peptides were synthesized and incubated with purified rSIRT2 protein for in vitro deacetylation under the conditions described previously.^[^ ^12^ ^]^ Purified rSIRT2 protein effectively deacetylated the acetyl‐peptides of these extracellular or matrix proteins with or without vWA domain (Figure 6B).

ITGB3 is the vWA domain‐bearing integrin, a well‐known driver of anchorage‐independent cell survival and metastasis.^[^ ^39^, ^40^, ^41^ ^]^ ITGB3 was acetylated on K416 within the vWA domain known to mediate ligand binding. To verify secreted SIRT2 targets ITGB3 for deacetylation in vivo, an antibody against ITGB3‐K416 acetylation was generated and validated with dot/slot blot (Figure 6C). The nonspecific SIRT inhibitor NAM or HDAC inhibitor TSA treatment greatly induced ITGB3 K416 acetylation in A549 cells (Figure 6D), suggesting both Sirtuin and HDAC‐type deacetylases could induce ITGB3 deacetylation. In HEK293T cells transiently co‐transfected with SIRT2 and ITGB3, SIRT2 and ITGB3 were co‐immunoprecipitated (Figure 6E). Meanwhile, ITGB3 transfection along with CBP also increased the ITGB3‐K416 acetylation level (Figure 6F), suggesting integrin acetylation could be dynamically regulated by endogenous acetyltransferase. Conversely, SIRT2 overexpression reduced ITGB3‐K416 acetylation levels (Figure 6F). Similarly, the deacetylation of SIRT2 was also found in Vitronectin (VTN), a major component of the ECM. VTN acetylation level was largely reduced when VTN was coexpressed with the wild type SIRT2, but not with the SIRT2 H187Y mutant (Figure S4A, Supporting Information). To further confirm transmembrane protein ITGB3 is deacetylated by eSIRT2 secreted from cells in microenvironment, ITGB3 expressing HEK293T cells were co‐cultured with SIRT2‐expressing HEK293T cells. Expression of SIRT2 in full length rather than these secretion‐deficient SIRT2 Δ340–345 induced ITGB3 K416 deacetylation (Figure 6G). Similarly, ITGB3‐K416 acetylation level was significantly higher in ITGB3‐expressing HEK293T cells treated with supernatants collected from SIRT2‐expressing ATG7‐KO cells than from wild type cells (Figure 6H). ITGB3‐K416 acetylation signal was mainly visualized on cell membrane or in the extracellular space of A549 cells (Figure 6I) but was markedly decreased when THP1 cells or LPS pretreated‐THP1 cells were incubated (Figure 6I).

To evaluate the catalytic activity of the autophagic SIRT2 on intracellular proteins, histone proteins prepared from cancer cells were incubated with the medium collected from THP1 cells treated with LPS, which contained secreted SIRT2. H3K9ac intensity was reduced in a time dependent manner (Figure S4B, Supporting information). Like histone deacetylation, ITGB3 extracellular domain deacetylation by autophagic SIRT2 was NAD^+^‐dependent. Given that SIRT2 is an NAD^+^‐dependent enzyme,^[^ ^42^ ^]^ a nuance change in the NAD^+^/NADH ratio could potentially affect SIRT2 catalytic activity.^[^ ^43^ ^]^ When cocultured with LPS‐activated macrophages, the decreased intracellular NAD^+^ level was accompanied by an NADH level increase (Figure S4C, Supporting Information). Therefore, secreted SIRT2 protein also utilizes NAD^+^ to catalyze deacetylation in microenvironment and autophagic SIRT2 proteins exhibited no discrepancy in extracellular and cytoplasmic protein deacetylation initiation.

### 2.5 Extracellular SIRT2 Facilitates Cancer Cell Invasion, Migration, and Metastasis

**Figure 7:**
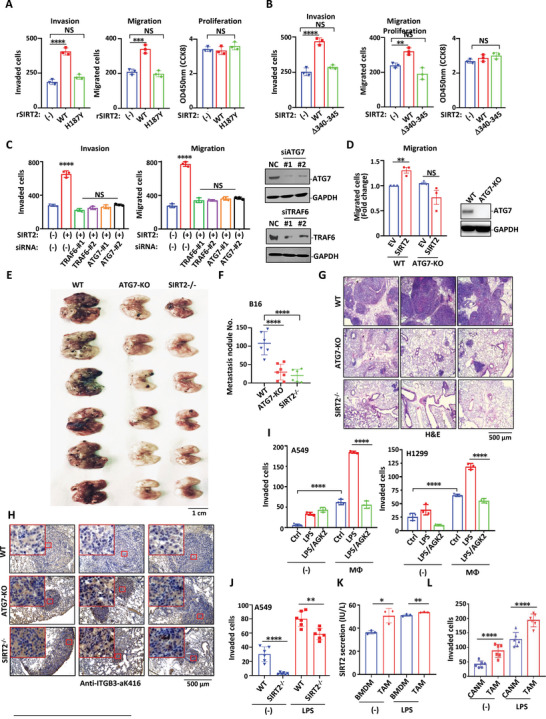
Secreted SIRT2 promotes lung cancer metastasis. A) Cell migration and invasion were assessed in A549 cells by Transwell assay using culture medium supplemented with purified rSIRT2 WT or rSIRT2 H187Y proteins as chemoattractant. Migrated or invaded A549 cells were stained with crystal violet. The proliferation of A549 cells was determined by CCK‐8 assay. B) A549 cell migration and invasion toward culture medium from either SIRT2 WT‐transfected or the SIRT2 D340–345‐transfected HEK293T cells were determined by Transwell assay. The proliferation of A549 cells was determined by CCK‐8 assay. C) Transwell assay of invasion and migration of A549 cells using culture medium from either SIRT2 WT transfected HEK293T cells, SIRT2 WT plus siTRAF6, or SIRT2 WT plus siATG7 co‐transfected HEK293T cells as chemoattractant. Western blot analysis confirmed the knockdown of TRAF6 and ATG7 in these cells. D) Transwell assay of migration of H1299 cells using culture medium from either empty vector‐transfected or SIRT2‐transfected WT or ATG7‐KO HEK293T cells as chemoattractant. Western blot analysis of ATG7 expression was shown on the right. E–H) B16 melanoma cells were injected into wild‐type, SIRT2^−/‐^ or ATG7‐KO mice through the tail vein. E) Representative images of whole lungs with B16 melanoma cell nodules. F) Metastatic nodules were counted 14 days post B16 cell tail injection. G) H&E staining of the lung sections of the above mice. H) The levels of ITGB3 K416 acetylation in lung cancer tissues of three types of animals as indicated were compared by IHC staining with the ITGB3‐aK416 antibody. I) Lung cancer cells (A549 cells and H1299 cells) were cultured alone or co‐cultured with macrophages treated with LPS or AGK2 plus LPS followed by transwell migration assay. J) A549 cells (2 × 10^5^) were cultured alone or co‐cultured with wildtype or SIRT2^−/−^ macrophages (2 × 10^5^) in the presence or absence of LPS. Transwell migration assay was performed to analyze metastasis tendency of these A549 cells. K) Lewis lung cancer cells (5 × 10^6^) were subcutaneously injected into the flank of C57BL/6 mice. 6 weeks later, the primary tumors were excised. 2 × 10^5^ TAMs isolated from the tumor tissues were compared with BMDMs for SIRT2 secretion in response to LPS or no treatment. Secreted SIRT2 were measured with ELISA. L) Lewis lung cancer cells (2 × 10^5^) were cocultured with macrophages isolated from lung cancer adjacent normal tissues (CANM) or lung tumor tissues (TAM) obtained from lung cancer patients, treated with or without LPS (2 µg mL^−1^) for 12 h. 24 h after incubation, Lewis lung cancer cells left in the Boyden chamber were stained with crystal violet and photographed. Columns represented mean ± SEM of 3 experiments. Data are shown as mean ± SEM. **, *p* < 0.01; ***, *p* < 0.001; ****, *p* < 0.0001; NS suggests no significant difference between mock‐treated (‐) and the indicated group.

The implication of SIRT2 in carcinogenesis and tumor progression remains controversial. For instance, there is a correlation between upregulation of SIRT2 with malignancy progression in hepatocellular carcinoma (HCC) and NSCLC.^[^ ^14^, ^44^, ^45^ ^]^ On the contrary, there is also evidence to support that SIRT2 deficiency in mice causes tumorigenesis in mammary tumors and HCC. Furthermore, human breast cancers and HCC samples exhibited reduced SIRT2 levels.^[^ ^46^, ^47^ ^]^ In this regard, we assessed the effects of extracellular SIRT2 proteins on lung cancer cell migration and invasion. Purified rSIRT2 wild type proteins but not rSIRT2‐H187Y mutant significantly increased A549 cell migration as well as invasion, but showed no effect on A549 cell proliferation (**Figure** **7**A and Figure S5A, Supporting Information). Similarly, A549 cells were incubated with conditioned medium collected from HEK293T cells expressing a wild type or a secretion‐deficient form of SIRT2. The conditioned medium of wild type SIRT2 significantly increased cancer cell invasion and metastasis properties without affecting the proliferation property in A549 cells (Figure 7B and Figure S5B, Supporting Information). Similar results were obtained with another lung adenocarcinoma cell line SPCA1 (Figure S5C,D, Supporting Information).

We have evaluated the effect of extracellular SIRT2 on tumor microenvironmental editing using integrin (*α*v*β*3) targeted Arg‐Gly‐Asp (RGD) peptide probe for cancer optical imaging.^[^ ^48^ ^]^ The vWAF domain of Integrin extracellular domain can bind to the RGD motif of matrix proteins.^[^ ^49^ ^]^ RGD peptide‐based probes have emerged as a promising approach for visualization, localization, and measurement of cancer cells in vivo. LPS‐activated THP1 cells or to a lesser extent, THP1 cells reduced RGD‐peptide association with A549 cells (Figure S5E, Supporting Information). As expected, RGD peptide treatment markedly blocked lung cancer cell metastasis (Figure S5F–I, Supporting Information). Although macrophages from different sources could all accelerate metastasis (Figure S5F–I, Supporting Information), RGD‐peptides exhibited an inhibitory effect on cancer cell metastasis (Figure S5F–I, Supporting Information). These results provide additional evidence for our conclusion that integrin extracellular domain acetylation plays a critical role in lung cancer cell metastasis. Integrin extracellular domain deacetylation by SIRT2 secreted by macrophages promotes cancer cell migration.

Further, the SIRT2‐induced enhancement in cell invasion and migration was abolished when Lewis cells were cocultured with autophagy inhibitor Chloroquine (CQ) pre‐treated TAMs (Figure S5H,I, Supporting Information), or A549 cells were treated with supernatants harvested from transient TRAF6‐ or ATG7‐knockdown HEK293T cells using siRNAs or from stable ATG7‐KO cells using CRISPR‐Cas9 (Figure 7C,D and Figure S5J,K, Supporting Information).

We next explored lung metastasis of cancer cell through intravenous injection of B16 melanoma cells into wild‐type, ATG7‐KO, or SIRT2^−/−^ mice. Consistent with a recent report,^[^ ^50^ ^]^ ATG7‐KO mice had markedly less melanin metastatic foci in lung. Similar effects were also observed in SIRT2^−/−^ mice, in which experimental lung metastasis was significantly diminished as compared to their wild‐type littermates (Figure 7E–G and Figure S5L, Supporting Information). Immuno‐histochemistry (IHC) staining revealed that ITGB3 vWA domain K416 acetylation was dramatically increased at lung B16 metastatic nodules in both SIRT2^−/−^ and ATG7‐KO mice (Figure 7H). In addition, Lewis lung cancer cells were subcutaneously injected alone or along with purified rSIRT2 proteins into the flank of male C57BL/6 mice. Purified rSIRT2 proteins strongly stimulated metastasis of these subcutaneously injected Lewis lung cancer cells into lung (Figure S5M, Supporting Information). Macrophages treated with LPS accelerated the metastasis of A549 or highly‐metastatic neuro‐endocrine H1299 lung cancer cells as assessed by a transwell migration assay. But macrophages pre‐treated with the SIRT2 inhibitor AGK2 effectively blocked LPS‐induced metastasis of the A549 or H1299 lung cancer cells (Figure 7I). Macrophage with SIRT2 knockout attenuated their effect on A549 cell metastasis in response to LPS treatment (Figure 7J). Although macrophages from different origins all can secret SIRT2, TAMs were more effective in SIRT2 secretion as compared with BMDMs here (Figure 7K). As expected, TAMs were more effective than macrophages obtained from the CANMs in metastasis induction (Figure 7L).

Orthotopic implantation of cancer was conducted but SIRT2^−/−^ mice only showed a mild increase in tumor growth when B16 cells were subcutaneously applied (Figure S5N,O, Supporting Information), implying a selective role of secreted SIRT2 in alleviating cancer metastasis than tumor growth in vivo. Blockage of SIRT2 secretion or inhibition of extracellular SIRT2 activity might be a promising therapeutic strategy in anti‐metastasis therapy.

### 2.6 Serum SIRT2 Levels and ITGB3‐K416 Deacetylation Positively Correlate with Poor Prognosis Human Lung Cancer

**Figure 8:**
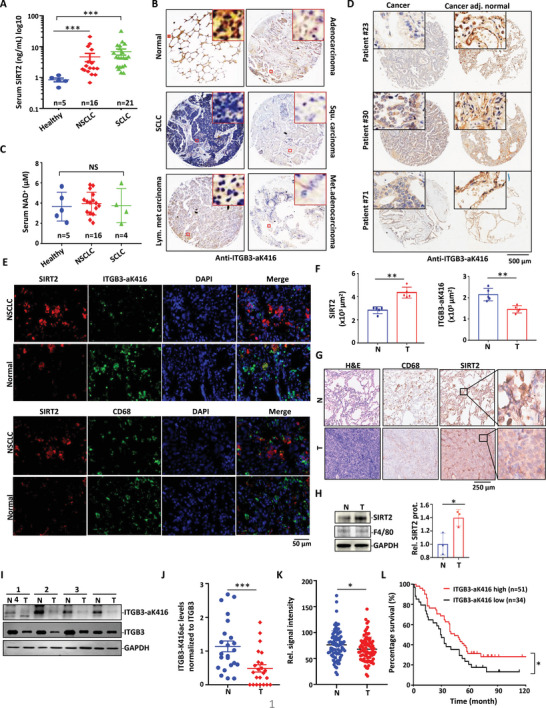
SIRT2 secretion and ITGB3 deacetylation correlate with poor prognosis in human lung cancer. A) Blood samples collected from healthy controls (*n* = 5), NSCLC patients (*n* = 16), and SCLC patients (*n* = 21) were analyzed for SIRT2 protein levels with ELISA. B) Lung tissues from normal people or patients with different types of lung cancer were subjected to IHC analysis for ITGB3 acetylation using ITGB3‐aK416 antibody. C) Blood samples collected from healthy controls (*n* = 5) and lung cancer patients including NSCLC (*n* = 16) and SCLC (*n* = 4) patients were analyzed for NAD+ levels. D) Lung adenocarcinoma and adjacent normal tissues from three patients were subjected to IHC analysis for ITGB3 acetylation. E,F) Immunofluorescence analysis of SIRT2, ITGB3‐aK416, and CD68 in lung adenocarcinoma and adjacent normal tissues. DAPI staining was used to label the nucleus. Intensity profiles were generated in different groups using ImageJ in (F). G) IHC analysis of CD68 and SIRT2 from coherent tissue. H&E staining of lung adenocarcinoma and adjacent normal tissues was also performed. H) Tumor associated macrophages (T) or normal tissue associated macrophages (N) (1 × 10^6^) used in Figure 7K were examined for SIRT2 protein expression with Western blot. Relative SIRT2 protein expression intensities from three experiments were quantitated with ImageJ. I,J) Lung tumor tissues (T) and adjacent normal tissues (N) were obtained from lung adenocarcinoma patients and subjected to Western blot analysis with antibodies against ITGB3‐K416 acetylation and ITGB3. Representative immunoblots are shown in (I). Densitometry of immunoblots was measured by ImageJ in (J). K) The IHC staining intensity scores for ITGB3‐K416 acetylation in lung cancer tissue microarray (*n* = 85). T: lung tumor tissues, N: adjacent normal tissues. L) Kaplan‐Meier analysis of overall survival in the above 85 patients according to ITGB3‐K416 acetylation intensity. Score ≤ 60 was considered as low while score > 60 as high. Data are shown as mean ± SEM. *, *p* < 0.05; ***, *p* < 0.001; NS suggests no significant difference between control and the indicated groups or between the indicated two groups.

To explore the association of SIRT2 secretion with ITGB3‐K416 deacetylation in human cancers, we collected blood samples from lung cancer patients. It was found that patients with NSCLChad significantly higher levels of eSIRT2 in serum than those of healthy individuals (**Figure** **8**A). Patients with small cell lung cancer (SCLC) showed even higher eSIRT2 levels than those of NSCLC (Figure 8A). This finding correlates with the immunohistochemical (IHC) staining finding that the ITGB3‐K416 acetylation level was much lower in lung tissues obtained from cancer patients than in healthy controls (Figure 8B). SIRT2 catalytic activity requires NAD^+^ as the cofactor. There were no apparent differences in the NAD^+^ levels in blood between healthy people and cancer patients (Figure 8C).

**Figure 9:**
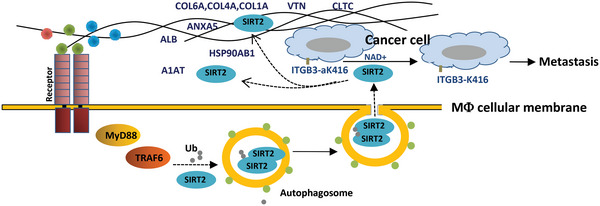
Illustration of promotion of lung cancer metastasis by SIRT2‐mediated extracellular protein deacetylation. Macrophages secrete SIRT2 protein into extracellular space where SIRT2 protein deacetylates proteins of cell membrane and extracellular matrix, resulting in cancer cell metastasis.

ITGB3 is tightly associated with lung cancer metastasis.^[^ ^39^, ^40^, ^51^ ^]^ We examined acetylation within the vWA domain of ITGB3 in lung adenocarcinoma tissues by a microarray‐based IHC, immunofluorescence, and Western blot analysis. ITGB3 K416 acetylation level was higher in adjacent normal tissues than that of tumor specimens as revealed by IHC staining(Figure 8D and Figure S6A, Supporting Information). Immunofluorescent staining revealed a higher level of SIRT2 proteins in NSCLC lung tumor tissues than normal tissues, which inversely correlated with a lower level of ITGB3‐aK416 in NSCLC tumor tissues than normal tissues (Figure 8E). Notably, immunofluorescent staining detected SIRT2 proteins either barely overlay with ITGB3‐aK416 positive lung cancer cells or sporadically overlay with CD68 positive macrophages, indicating SIRT2 proteins visualized here were mainly distributed in the extracellular space (Figure 8E,F). Consistently, IHC staining also exhibited an extracellular distribution pattern of SIRT2 proteins in NSCLC tumor tissue (Figure 8G). Tumor‐associated macrophages seemed to express a higher level of SIRT2 protein than non‐tumor associated macrophages (Figure 8H). ITGB3 K416 acetylation intensity was significantly higher in cancer tissue samples than that in normal tissue samples obtained from lung cancer patients (Figure 8I,J and Figure S6B, Supporting Information). Collectively, SIRT2 proteins secreted from macrophages were responsible for ITGB3 aK416 deacetylation of lung cancer cells. Despite SIRT2 may also deacetylate other extracellular proteins such as collagens, we failed to detect any apparent difference in the acetylation intensity of COL6A2 at K185 between tumor tissue and normal tissue (Figure S6C, Supporting Information). Clinical characteristics of lung cancer patients were summarized in Figure S6D, Supporting information. However, Meta‐analysis from several lung adenocarcinoma studies revealed that the overall SIRT2 level exhibited no apparent difference between cancer and normal tissue (Figure S6E, Supporting Information). The intensity scores of lung cancer tissues versus adjacent normal tissues from the microarray‐based IHC were calculated and a significant difference was observed between them (Figure 8K). We divided these tissues into high and low levels of ITGB3‐K416 acetylation groups according to the IHC intensity scores. Low levels of ITGB3‐K416 acetylation were found to be significantly associated with poor outcomes in patients with lung adenocarcinoma (Figure 8L). Therefore, a close connection between SIRT2 secretion from macrophages and ITGB3 vWA domain (K416) deacetylation in the tumor microenvironment of human lung cancer patients has been established (**Figure** **9**).

## 3 Discussion

It is widely known that most intracellular proteins undergo reversible post‐translational modifications within cells. But how extracellular proteins or extracellular domains of transmembrane receptors undergo reversible post‐translational modifications remains largely unknown. Reversible acetylation has emerged as an important regulation of protein activities involved in transcription factors and epigenetic gene regulation in nuclei, signal transduction, and metabolism regulation in cytoplasm and mitochondria. Acetylation and deacetylation also play a critical role in the extracellular space to trigger cell migration or metastasis. Sirtuin family members have been assigned to different places of the cells to regulate cellular activity via deacetylation induction. While SIRT1, SIRT6, and SIRT7 translocate into nuclei to regulate gene transcription, SIRT3, SIRT4, and SIRT5 translocate to the mitochondria to participate in metabolic regulation. SIRT2 however undergoes extracellular space translocation.

While the N‐terminal signaling peptide carrying proteins are released through the ER‐Golgi canonical secretion route, proteins without that N‐terminal signaling peptide can be secreted via pathways including secretory autophagy.^[^ ^29^ ^]^ Protein kinase VLK and phosphatase PTEN can be constitutively secreted via their N‐terminal signaling peptide guidance in cancer cells.^[^ ^8^, ^9^, ^10^ ^]^ SIRT2 can be secreted from macrophages under stimulation. The “ELEDLV” motif within the C‐terminal domain sandwiched by positively charged residues dominates SIRT2 secretion. One of the best characterized examples of autophagosome‐mediated secretion is IL‐1*β*. IL‐1*β* is sequestered into autophagosomes and delivered to the extracellular space upon TLR activation.^[^ ^19^, ^52^, ^53^ ^]^ The secretory motif “ELEDLV” of SIRT2 is highly similar to the secretory motif “LQLESVD” of IL‐1*β*. The fact that TLR4 or TLR2 but not TLR3 activation can facilitate SIRT2 secretion excludes the MyD88‐independent pathway, that is, TRAM/TRIF‐IRF3 pathway in SIRT2 secretion for TLR3 activates MyD88‐independent pathway only. However, in the MyD88‐dependent pathway, TRAF6 plays a steering wheel‐like role to trigger apoptosis, survival, and/or autophagy. LC3‐I converts into LC3‐II in ATG7‐dependent manner and LC3‐II in turn integrates into the autophagosome vesicle body membrane. Ectopic expression of TRAF6 alone is sufficient for LC3‐I to LC3‐II conversion, suggesting the MyD88‐TRAF6 pathway facilitates autophagosome vesicle body formation.^[^ ^35^ ^]^ This suggests that the E3 ligase TRAF6 is the critical checkpoint for either autophagy flux induction or for TAK/TAB activation to free NF‐kB for nuclear translocation and gene expression regulation. The negatively charged and hydrophobic “ELEDLV” and “LQLESVD” sequences likely represent the secretion motif for SIRT2 or IL‐1*β* autophagosome translocation and subsequent secretion into the microenvironment. The secretion sequences are distinct from the nuclear exporting sequences (NES) because they include multiple negatively charged residues. Hence, the secretion motif carriers drive their secretion in an autophagosome‐dependent manner by macrophages.

The extracellular spaces or microenvironments can be considered as the subcellular organelles, similar to mitochondria. They serve as the platform for extracellular proteins to undergo post‐translational modifications and perform their functions. Among the extracellular proteins of the acetylome as putative SIRT2 substrates identified with TMT mass spectrometry, the vWA domains of extracellular proteins mediate cell adhesion.^[^ ^54^ ^]^ Integrins bind to the “RGD” or “LDV” motif of fibronectins,^[^ ^55^ ^]^ whereas the vWA domains of collagens and integrins may form the interface between the vWA domains and the rest of the proteins involved in the interaction.^[^ ^56^ ^]^ SIRT2 deacetylates the vWA domain of ITGB3 to restore the positive charges of *ε*‐amines of lysine residues. The cellular membrane protein‐protein interactions were therefore dissociated to free the cancer cells for migration. SIRT2 also deacetylates extracellular proteins lacking the vWA domain including COL4A2 and COL1A. COL4A2 acetylation was increased in cells treated with aspirin,^[^ ^57^ ^]^ a well‐known anti‐cancer agent.A1AT is the most abundant proteinase inhibitor in extracellular space. A1AT protein from human serum was found acetylated on K298 site.^[^ ^6^ ^]^ In this work, we provided evidence that the orthologous K292 of mouse A1AT protein is the SIRT2‐deacetylation site. Like collagens, the Annexin family member ANXA5 also provides integrin as a binding partner in extracellular space. In humans, ANXA5 K101 was acetylated.^[^ ^58^ ^]^ Mouse ANXA5 K99 (the orthologous K101 of human ANXA5) is now shown to be a SIRT2‐deacetylation site. All of these extracellular proteins exert pro‐tumor activity especially metastasis.^[^ ^59^ ^]^ In this study, we demonstrated that the acetyl‐extracellular proteins could be deacetylated by secreted SIRT2. This provides a new scenario for the deacetylation‐dependent regulation of microenvironment.

Although the exact effect of SIRT2 in cancer development is still under debate, our study supports the role of secreted SIRT2 in promoting cancer cell detachment and migration, rather than growth. Dramatically elevated serum SIRT2 level in lung cancer patients especially these highly metastatic SCLC patients strongly correlated with deacetylation of ITGB3 vWA domain‐K416 as well as other extracellular proteins of cancer cells. Cancer cell deacetylation of ITGB3‐vWA domain‐K416 was correlative with a poor prognosis. Thus, aberrant SIRT2 secretion from macrophages contributes significantly to cancer metastasis. Our meta‐analysis found either no difference in SIRT2 expression between normal and lung cancer samples, nor correlation between SIRT2 expression and overall survival of lung cancer patients. This indicates that SIRT2 in extracellular space is more important than SIRT2 within the cells in terms of cell migration.

While SIRT family members can deacetylate cytoplasm and nuclear proteins with redundancy, SIRT2 is the only one found to fulfill extracellular deacetylation activity due to its ability to carry the IL‐1*β* secretion motif like sequence. Recently, an increasing number of studies have characterized inhibitors of SIRT2 as potential anti‐cancer drugs.^[^ ^60^, ^61^ ^]^ We expect that targeting eSIRT2 in extracellular space could be an interesting anti‐cancer therapy approach.

## 4 Experimental Section

### Animals and Patient Samples

SIRT2^−/‐^ mice were kind gifts from Prof. CX Deng (University of Macau). TLR4^−LPS‐del^ and TLR2^−/−^ mice were kindly provided by Prof. S Xiong (Soochow University). LysM‐Cre; ATG7^flox/flox^ mice were gifted from Prof. W. Chen (Zhejiang University). Sex‐ and age‐matched animals aged 6–8 weeks were used in all experiments unless otherwise specified. Mice were housed in the specific‐pathogen‐free facility at the Soochow University. All animal experiments were approved by the Animal Ethics Committee of Soochow University (SUDA20180905A01). Peripheral blood samples were obtained from consenting healthy volunteers, or NSCLC and SCLC patients at the First Affiliated Hospital of Soochow University, with approval by the Institutional Review Board of Soochow University (SUDA20180905H02). The study was performed in accordance with the Declaration of Helsinki. All subjects in study provided written informed consent.

### Cells and Cell Cultures

THP1 cells, RAW 264.7 cells, and HEK 293T cells were purchased from ATCC. Immortalized Bone‐marrow derived macrophages were prepared as previously described.^[^ ^62^ ^]^ Cells were maintained in DMEM or RPMI1640 containing 10% FBS and 1% Pen/Strep. For secretion assay, cells were stimulated with indicated stimuli in serum‐free medium. Stimuli were applied in the following concentration unless otherwise indicated: LPS (100 ng mL^−1^), Pam_3_CSK_4_ (100 ng mL^−1^), PGN (10 µg mL^−1^), PolyI:C (50 µg mL^−1^), IFNγ (10 ng mL^−1^) or IL‐4 (10 ng mL^−1^).

### Bead Halo Assay

Protein A‐agarose beads were incubated with SIRT2 antibody at a ratio of 1 µg antibody per 5 µL beads for 2 h in PBS, and incubated with 50 ng mL^−1^ PMA‐primed THP1 cells. The culture mixture was then treated with LPS (100 ng mL^−1^) for indicated times and visualized under fluorescence microscopy. In this assay, secreted GFP‐SIRT2 proteins in culture medium were captured by antibody coated beads.

### Adhesion and Competition Assays

Lung cancer cells were seeded into 12‐well Boyden Chamber. For the competition assay, cancer cells were incubated with the synthetic RGD peptide (final concentration, 2 µm; QYAOBIO, Shanghai) for 24 h at 37 °C. Cells that migrated through the pores were fixed with 4% paraformaldehyde and stained with 0.1% crystal violet and photographed. For immunofluorescence imaging, lung cancer cells were seeded into Nunc Lab‐Tek II Chamber Slide (Thermo Scientific) and incubated with RGD peptide conjugated with Biotin (2 µm) for 24 h. After fixation, cells were labeled with Avidin‐Alexa Fluor 647 and cell fluorescence was examined with confocal microscope (Leica TCS SPE).

### TCA Precipitation

Culture medium was collected and mixed with 1/5 volume of 100% (w/v) TCA. The mixture was incubated on ice for 30 min and centrifuged at 14 000 × *g* for 15 min at 4 °C. Protein pellets were washed twice with ice‐cold acetone, resuspended in 1× SDS sample buffer, and subjected to western blot analysis.

### Purification of rSIRT2 Protein for In Vitro Deacetylation Assay

SIRT2 was cloned into pET28a vector containing an N‐terminal hexahistidine (His) tag, and was transformed into E. coli BL21 (DE3). Isopropyl‐*β*‐D‐1‐thiogalactopyranoside (IPTG, 0.2 mm) was used to induce SIRT2 protein expression and the culture was grown for 18 h at 20 °C. The His‐tag SIRT2 protein was extracted and purified using a Ni‐NTA (QIAGEN, Alameda, CA) column, followed by desalination with Amicon Ultra‐15 centrifugal filter (EMD Millipore Corporation, USA). The in vitro deacetylation of acetyl‐peptides was performed by incubating purified SIRT2 (0.1 µg) with acetyl‐peptides (300 ng) in the deacetylation buffer (50 mm Tris‐HCl, pH8.0, 137 mm NaCl, 2.7 mm KCl, 1 mm MgCl2, 1 mg mL^−1^ BSA, 3 mm NAD+) at 37 °C for 2 h. The peptides were then desalted by Strata X C18 SPE column and analyzed by a Bruker DaltonicsUltrafleXtreme MALDI TOF/TOF instrument. Acetyl‐peptides used were as followed: A1AT, K292, KELISK(ac)FLLNR; ANXA5, K99, LKHALK(ac)GAGTD; ITGB3, K416, SCMGLK(ac)IGDTV; Clathrin heavy chain 1 (CLTC, K1441), VNYFSK(ac)VKQLP; COL4A2, K181, KEDRDK(ac)YRGEP; COL6A2, K135, RASFTK(ac)SLQGI; Heat shock protein 90β (HSP90B1, K577), DKKVEK(ac)VTISN; Serum albumin (ALB, K236), GERAFK(ac)AWAVA.

### CRISPR‐Cas9 Gene Targeting

Gene targeting by CRISPR‐Cas9 was accomplished by inserting gene‐specific sgRNAs into the pLentiCRISPR v2 vector. The sgRNAs were used as followed: mouse ATG7: GTCCAGGGCACTATTAAAGG; ATG *12*: AATGGGCTGTGGAGCGAACC; ATG14: CACAGACCCATCTTCCAGAG; FIP200: TCAAGATAGACCCAATGATG; human ATG7:^[^ ^63^ ^]^ ACACACTCGAGTCTTTCAAG; scramble:^[^ ^63^ ^]^ GCACTACCAGAGCTAACTCA. The constructs were prepared as lentiviral particles by transfection of HEK293T cells with packaging plasmids psPAX2 and pMD2.G. Cells were selected with puromycin for at least 2 weeks, and single colonies were expanded and validated by western blot analysis.

### Immunoprecipitation and Western Blot Analysis

For immunoprecipitation, cells were lysed in lysis buffer (20 mm Tris‐HCl, pH 7.4, 100 mm NaCl, 1% Triton X‐100, 1 mm EDTA, 10% Glycerol, 1 mm DTT, and complete protease inhibitor cocktail) on ice. Cell lysates were incubated with M2 beads or HA antibody‐conjugated beads for 3–5 h at 4 °C. Beads were then boiled in SDS sample buffer and subjected to SDS‐PAGE, before being transferred onto nitrocellulose membranes followed by primary antibody incubation for 1–3 h and secondary antibody (DyLight 680 or DyLight 800 conjugated) incubation for additional 1–3 h at 4 °C. Protein bands were visualized using Odyssey Infrared Imaging System (LI‐COR Biosciences, USA), and were analyzed by Image Studio 3.1 software.

### Flow Cytometry

Primary peritoneal macrophages after stimulations were stained with PE conjugated anti‐mouse CD86 and PE/Cy7 conjugated anti‐mouse CD206 for 1 h. To determine cell surface protein acetylation, lung cancer cells were stained with Alexa Fluor 647 conjugated pan acetyl‐K antibody. The stained cells were then analyzed on a BD FACS Canto II flow cytometer (BD Biosciences, USA), and data were analyzed with FlowJo software.

### ELISA

Sandwich ELISA kits (Cat# SEA430Hu for human and SEA430Mu for mouse) were used to measure secreted SIRT2 protein levels in human serum or in culture medium following the manufacturer's protocol (Cloud‐Clone Corp, USA).

### Measurement of Intracellular NAD^+^/NADH Level

Cancer cells were treated as indicated and intracellular NAD^+^ level was measured using NAD^+^/NADH assay kit (WST‐8) (Beyotime, S0175). First, extract samples from cells with extraction buffer and deproteinize with spin column. For NADH measurement, heat samples to 60°C for 30 min to decompose NAD^+^ and cool it on ice. Then, add samples and reaction mixture to wells and incubate for 5 min at room temperature to convert NAD^+^ to NADH. Finally, add NADH developer and incubate for 1–4 h while reaction cycles and analyze it with microplate reader multiple times (450 nm).

### In Vitro Ubiquitination Assay

In vitro ubiquitination assay was performed as previously described.^[^ ^64^ ^]^ Briefly, a reaction mixture containing recombinant E1 (80 nm), UbcH5c (1 µm), ubiquitin (50 µm), TRAF6 (20 nm), and SIRT2 (10 nm) was reconstituted in 20 µL ATP buffer (50 mm Tris‐HCl, pH 7.5, 2 mm ATP, 5 mm MgCl_2_, and 0.1 mm DTT). The mixture was incubated at 30 °C for 30 min, stopped by addition of SDS sample buffer and boiled at 95 °C for 5 min, and subjected to western blot analysis.

### Transmission Electron Microscope Analysis

Mouse peritoneal macrophages were fixed with 2.5% glutaraldehyde in PBS for >4 h and washed by PBS. Cells were postfixed with 1% osmium tetroxide and dehydrated with grading ethanol and acetone. Cells were then infiltrated with spurrs’ resin by graduate concentration series in acetone (50% for 1 h, 75% for 3 h, and 100% overnight), and polymerized at 70 °C for 9 h. Ultrathin‐sectioning was done using an Ultracut UCT ultramicrotome (Leica), and were stained by uranyl acetate and alkaline lead citrate for 15 min respectively and subjected to transmission electron microscope analysis on a HITACHI‐H‐7650 electron microscope.

### Immunofluorescence Analysis

Cells were fixed with 4% paraformaldehyde and permeabilized with 1% Triton X‐100, followed by blocking with 3% BSA for 1 h at 37 °C. Cells were then incubated with the primary antibody in 1% BSA‐PBS overnight at 4 °C, followed by staining with Alexa Fluor 488 or Alexa Fluor 555 conjugated secondary antibody for 45–60 min at room temperature. Nuclei were counterstained with Hoechst 33342 or DAPI. Images were acquired on a confocal microscope (NikonA1or Zeiss 880). Non‐permeable immunostaining was performed similarly as aforementioned but without fixation with 4% paraformaldehyde. Fluorescence intensity was determined by Image J software.

### TMT‐Based Mass Spectrometry

Lung or liver tissues harvested from mice were snap‐frozen in liquid nitrogen and lysed with sonication. Proteins were denatured, reduced, alkylated, and subjected to trypsin digestion. Tryptic peptides were desalted and labeled using 6‐plex TMT kit (ThermoFisher) according to the manufacturer's protocol. Peptides from different mouse tissues were labeled in the following way: WT‐liver 128, KO‐liver 129, WT‐lung 129, KO‐lung 130. To enrich peptides bearing acetylated lysine, tryptic peptides were incubated with pre‐washed antibody beads (PTM Biolabs) at 4 °C overnight, and the bound peptides were eluted from the beads with 0.1% TFA. The eluted fractions were combined, vacuum‐dried, and cleaned with C18 ZipTips (Millipore), followed by LC‐MS/MS analysis using Q Exactive Plus hybrid quadrupole‐Orbitrap mass spectrometer (ThermoFisher Scientific, USA).

The peptides were subjected to NSI source followed by tandem mass spectrometry (MS/MS) in Q Exactive Plus (ThermoFisher Scientific, USA) coupled online to the UPLC. Intact peptides were detected in the Orbitrap at a resolution of 70 000. The resulting MS/MS data were processed using MaxQuant with integrated Andromeda search engine (v.1.4.1.2). Tandem mass spectra were searched against Swissprot_mousedatabase (16 717 sequences) concatenated with reverse decoy database. Trypsin/P was specified as cleavage enzyme allowing up to 5 missing cleavages, 5 modifications per peptide, and 5 charges. Mass error was set to 10 ppm for precursor ions and 0.02 Da for fragment ions. False discovery rate (FDR) thresholds for protein, peptide, and modification site were specified at 1%. The minimum peptide length was set at 7. The site localization probability was set as > 0.75. The quantified proteins in this study were divided into three quantiles by setting a quantification ratio of >1.2 as up‐regulated threshold and <0.83 as down‐regulated threshold. The *p*‐value (*x*) and fold change (*y*) of the Log2 L/H ratios of all quantified peptides were calculated. Gene Ontology (GO) annotation proteome was derived from the UniProt‐GOA database (http://www.ebi.ac.uk/GOA/). Wolfpsort version PSORT/PSORT II was used to predict subcellular localization.

### Migration, Invasion, and Proliferation Assays

Human lung cancer cells in serum‐free medium were seeded in the upper chamber with an 8.0 µm pore (Corning, USA) with (invasion) or without (migration) Matrigel (BD Bioscience, USA). Indicated cells or conditioned medium harvested from indicated cells were added to the lower chamber. Cells that migrated through the pores were fixed with 4% paraformaldehyde and stained with 0.1% crystal violet. Cell proliferation was measured using Cell Counting Kit‐8 assay (Dojindo Molecular Tech Inc, Japan) following the manufacturer's instructions.

### Generation of Acetylated ITGB3 Antibody

The modified peptide was first synthesized as follows. ITGB3 (K416):SCMGLK(ac)IGDTV.ITGB3 (K416)‐control: SCMGLKIGDTV. For effective immunization and generation of antibodies, the peptide was then conjugated to carrier protein keyhole limpet hemocyanin as described. When immunizing, the back of rabbits was immunized subcutaneously. The immune points of each rabbit were more than 4 points and the area near the neck was given priority to ensure immunization, following the immune cycle of 2:2:1:1:1 week. Finally, the serum of rabbits was collected and purified through polypeptide coupling column. It was achieved by automatic numerical control antibody purification machine. The machine was complete, in turn coupling medium cleaning, adding activator, cleaning the medium, coupling peptide, removing residual polypeptide, closing the active sites of medium. Adsorption chromatography of serum was finished in control peptide reaction tube and chromatographic adsorption of circulating liquid was repeated by modifying peptide reaction tube again. After rinsing three times, antibody was dissociated with addition of acid and alkali solution and the dissociation solution automatically formed a neutral phosphate solution after mixing. Purified antibodies were stored in antibody collection tubes.

### Metastasis Mouse Model

B16 cells (4 × 10^6^) in 100 µL PBS were injected into mice intravenously via tail veins. On day 16, the mice were euthanized and lungs were harvested after perfusion through the right ventricle of the heart with PBS. Cancer cell metastasis was evaluated by counting the number of visible dark nodules on the lung tissues. Lung tissues were then fixed in 4% PFA and embedded in paraffin blocks for histological analysis.

Orthotopic implantation of lung cancer was conducted as described. Mouse lung cancer cells Lewis (5 × 10^6^) were subcutaneously injected alone or along with purified rSIRT2 protein into the flank of male C57BL/6 mice. Primary tumors were surgically removed when they reached a volume of ≈200 mm^3^ to eliminate the effect of primary tumor size on metastasis occurrence. Mice were killed at 8 weeks after primary tumor removal, and lung metastasis was examined by routine histopathological analysis. All mouse experiments were approved by the Institutional Animal Care and Use Committee of Zhejiang Provincial People's Hospital.

### H&E Staining and Immunohistochemistry Analysis

Paraffin embedded lung tissues were cut into 5 mm sections and stained with H&E according to routine protocols. Standard IHC staining was performed as described elsewhere.^[^ ^65^ ^]^ Tissue microarray sections used in this paper comprised of 85 matched pairs of lung adenocarcinoma and adjacent normal tissue specimens. Tissue sections were incubated sequentially with ITGB3‐aK416 primary antibody overnight at 4 °C and horseradish peroxidase conjugated secondary antibody 1 h at room temperature, followed by immersed in DAB‐peroxidase substrate solution and counterstained with hematoxylin. Histological slides were scanned with a slide scanning instrument (DMetrix).

### Statistical Analysis

Statistical analysis of data was performed by student's *t*‐test or ANOVA test using GraphPad Prism 8.0 software. All data are expressed as mean ± SEM and values of *p* < 0.05 were considered to represent a significant difference statistically. Statistical significance is reported as follows: ns, not significant; * *p* < 0.05; ** *p* < 0.01; *** *p* < 0.001, **** *p* < 0.0001. Bars represent means ± SEM.

## Conflict of Interest

The authors declare no conflict of interest.

## Author Contributions

M.W., J.‐B.Z., Y.‐W.X., and Y.‐X.Z. contributed equally to this work. M.W., M.‐G.Z., Y.‐W.X., H.‐B.L., and Y.E.C. designed the experiments. M.‐G.Z., M.W., Y.‐W.X., Y.‐X.Z., Y.‐Y.Z., X.‐J.C., N.‐N.C., C.H., and X.L. performed most of the experiments including cell cultures, cloning genes, and flow cytometry. L.H. preformed ubiquitination analysis. Y.‐X.Z. and M.W. performed mass spectrometry analysis. X.m.L., J.‐C.Y., and M.W. performed the bioinformatics. Y.‐Y.Z., W.‐X.W., Q.‐H.Y., F.H., and J.‐B.Z. performed lung tissue staining and provided lung cancer samples. M.‐G.Z., Z.‐J.C., X.‐L.H., X.‐X.W., W.‐J.F., and J.‐B.Z. performed the animal studies. M.W., Y.‐W.X., M.‐G.Z., J.D., T.C.Z., H.‐B.L., and Y.E.C. wrote the manuscript with contributions from all authors.

## Supporting information

Supporting Information

## Data Availability

The data that support the findings of this study are openly available in pubmed, reference number 1234567890.
