# Supplementary material for: Promotion of Lung Cancer Metastasis by SIRT2‐Mediated Extracellular Protein Deacetylation
Source: Adv Sci (Weinh). 2022 Dec 1;10(3):2205462. doi: 10.1002/advs.202205462 (PMC9875677; doi:10.1002/advs.202205462)
Supplement: Supplementary file 1 — Supporting Information [file ADVS-10-2205462-s001.pdf]

ADVANCED SCIENCE
Open Access

Supporting Information

for *Adv. Sci.*, DOI 10.1002/advs.202205462

Promotion of Lung Cancer Metastasis by SIRT2-Mediated Extracellular Protein Deacetylation

*Meng Wu, Jian-Bin Zhang, Yi-Wei Xiong, Yong-Xu Zhao, Meng-Ge Zheng, Xia-Li Huang, Fang Huang, Xing-Xing Wu, Xue Li, Wei-Jiao Fan, Lin Hu, Yuan-Yuan Zeng, Xia-Ju Cheng, Ji-Cheng Yue, Juan-Juan Du, Nan-Nan Chen, Wen-Xiang Wei, Qing-Hua Yao, Xiao-mei Lu, Chao Huang, Jiong Deng, Zhi-Jie Chang, He-Bin Liu, Ting C. Zhao and Y. Eugene Chinn**

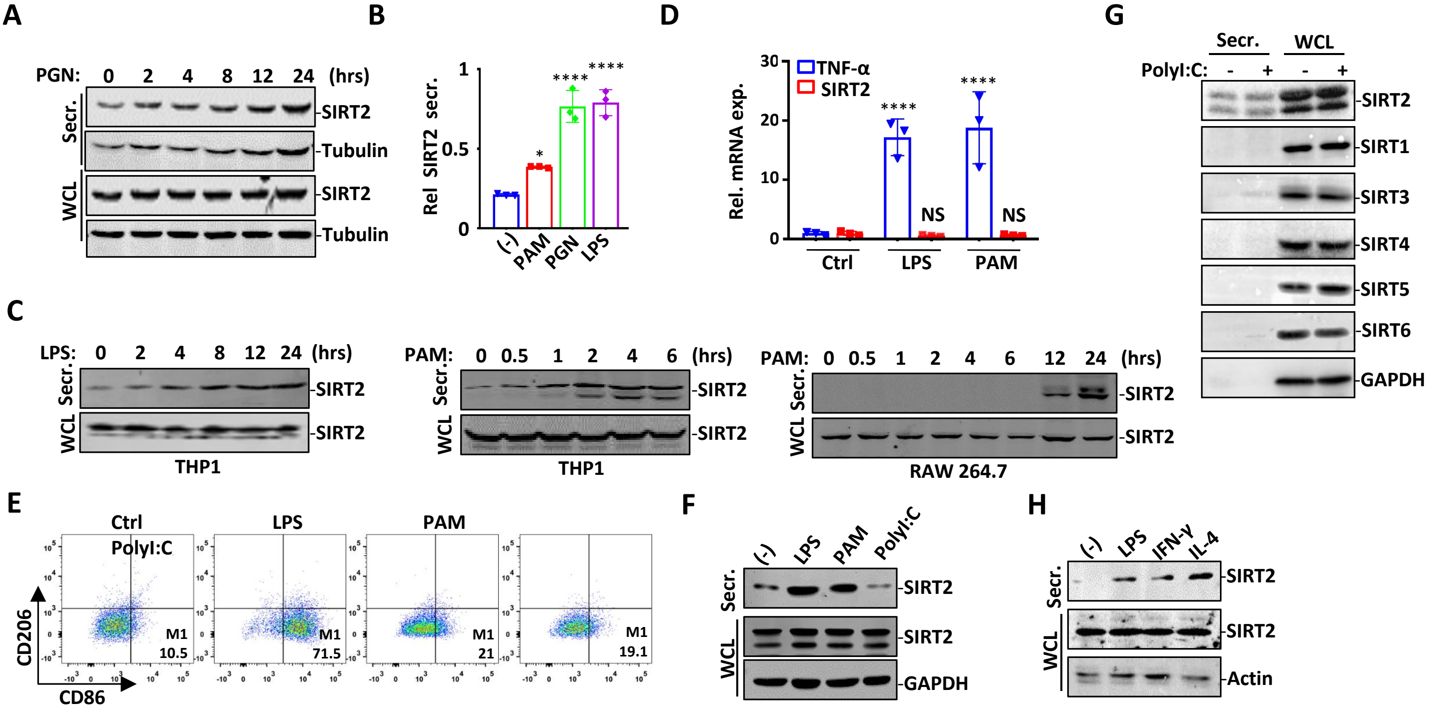

Figure S1. Macrophages secrete SIRT2 in response to multiple stimuli, related to Figure 1. (A) Mouse peritoneal macrophages were treated with PGN for a time course up to 24 hrs, SIRT2 proteins from Secr. and WCL fractions were analyzed in Western blot with SIRT2 antibody. Secreted Tubulin proteins were also detected. **(B)** SIRT2 proteins secreted from mouse macrophages in response to indicated TLR ligands for treatment were analyzed with ELISA for SIRT2 secretion. Data are mean ± SEM. *, *p*<0.05; ***, *p*<0.001; NS, no significant difference between control and the indicated groups. **(C)** PMA-primed THP1 cells or Raw 264.7 cells were treated with LPS or PAM for indicated time, Secr. or WCL samples were analyzed with Western blot using SIRT2 antibody. **(D)** Mouse peritoneal macrophages were treated with LPS, PAM or left unstimulated for 24 hrs. TNFα and SIRT2 mRNA expression levels were determined by RT-qPCR . Data are mean ± SEM. ***, *p*<0.001; ****, *p*<0.0001; NS, no significant difference between control and the indicated groups. **(E)** Mouse peritoneal macrophages were treated with the indicated agents for 24 hrs. Macrophage polarization was analyzed with CD86 and CD206 antibodies by flow cytometry. **(F and H)** Representative blots of mouse peritoneal macrophages treated with different ligands as indicated for 24 hrs in (F) and (H). SIRT2 abundance in cell supernatant (Secr.) and WCL samples were analyzed in Western blot with SIRT2 antibody or GAPDH/β-actin antibody. **(G)** Mouse macrophages were treated with PolyI:C or left unstimulated for 24 hrs. Secr. and WCL fractions were analyzed with Western blot using antibodies against SIRT family members as indicated.

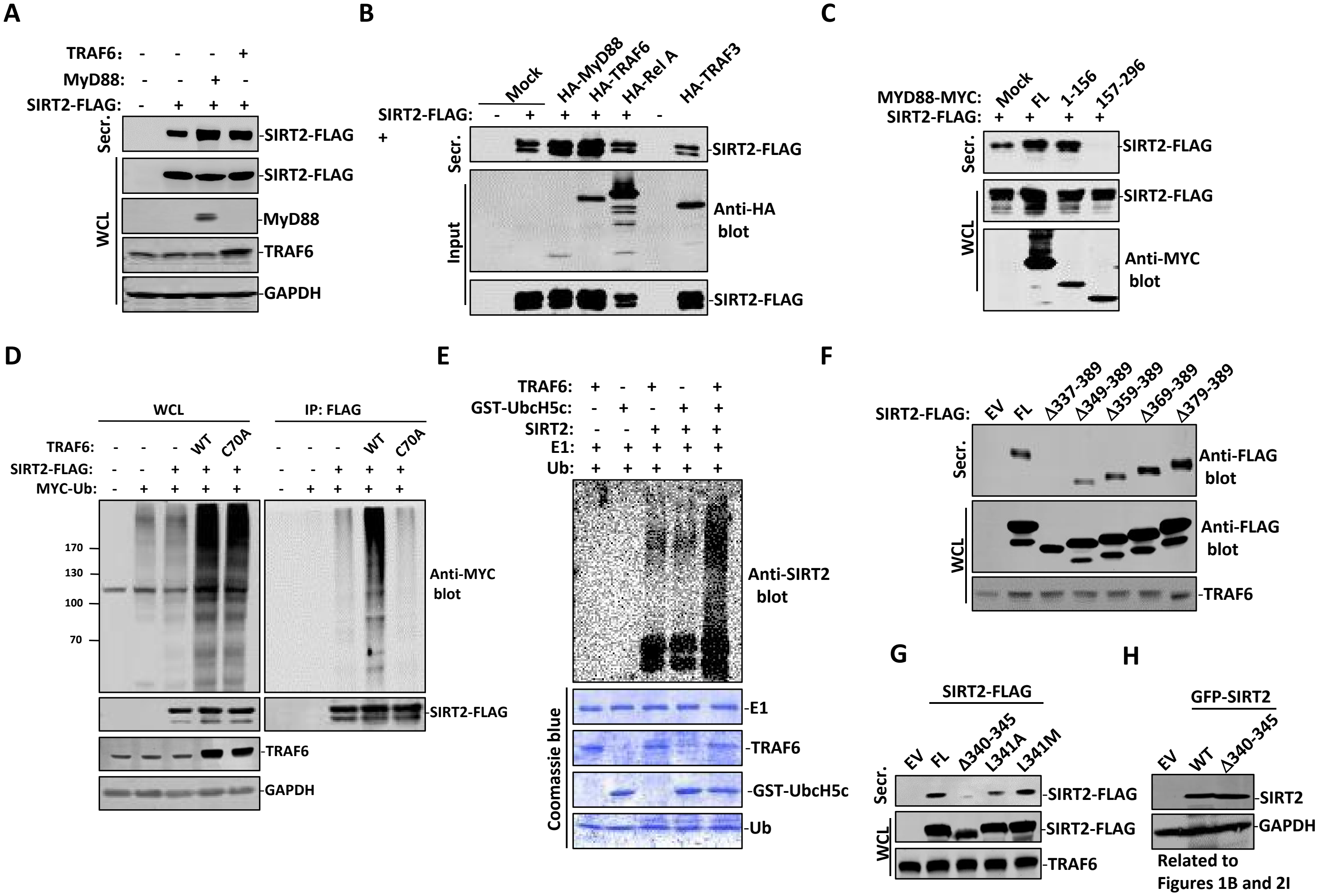

Figure S2. TRAF6-E3 ligase mediates SIRT2 secretion, related to Figure 2. (A) SIRT2-Flag was co-transfected with EV, MyD88 or TRAF6 in HEK293T cells. Secreted SIRT2-FLAG were enriched with anti-FLAG M2 agarose beads and eluted with sample buffer. WCL and eluate were subjected to Western blot analysis using antibodies as indicated. **(B)** SIRT2-Flag was co-transfected with HA-MyD88, HA-TRAF6, HA-RELA or HA-TRAF3 into HEK293T cells. Secreted SIRT2-Flag (enriched with M2 beads) and whole cell lysate were subjected to Western blot using Flag and HA antibodies. **(C)** SIRT2-Flag was co-transfected with MyD88 full-length (FL), or 1-156, 157-296 truncation mutants in Myc tagged form into HEK293T cells. Secreted SIRT2-Flag (enriched with M2 beads) and WCL were subjected to Western blot using Flag and Myc antibodies for analysis. **(D)** HEK293T cells were transfected with Myc-Ub, SIRT2-Flag, TRAF6 WT and TRAF6 C70A as indicated. WCL was collected and subjected to immunoprecipitation with anti-Flag antibody followed by Western blot with Myc or Flag antibody. **(E)** E1, UbcH5c (E2), His-TRAF6, Ub and His-SIRT2 were purified from bacteria. Recombinant proteins were incubated in combinations as indicated, followed by Wester blot analysis using SIRT2 antibody and Coomassie Blue staining in parallel. **(F)** Various C-terminal domain deletion mutants of SIRT2-FLAG were transiently expressed in HEK293T cells as indicated. Secreted SIRT2-Flag proteins were analyzed by Western blot with Flag antibody. **(G)** L341A and L341M mutants of SIRT2-Flag were transiently expressed in HEK293T cells. Secreted SIRT2-Flag and WCL were analyzed by Western blot with Flag antibody. **(H)** Expression of ectopic GFP-SIRT2 WT and GFP-SIRT2 Δ340-345 in THP1 cells were confirmed by Western blot with SIRT2 antibody

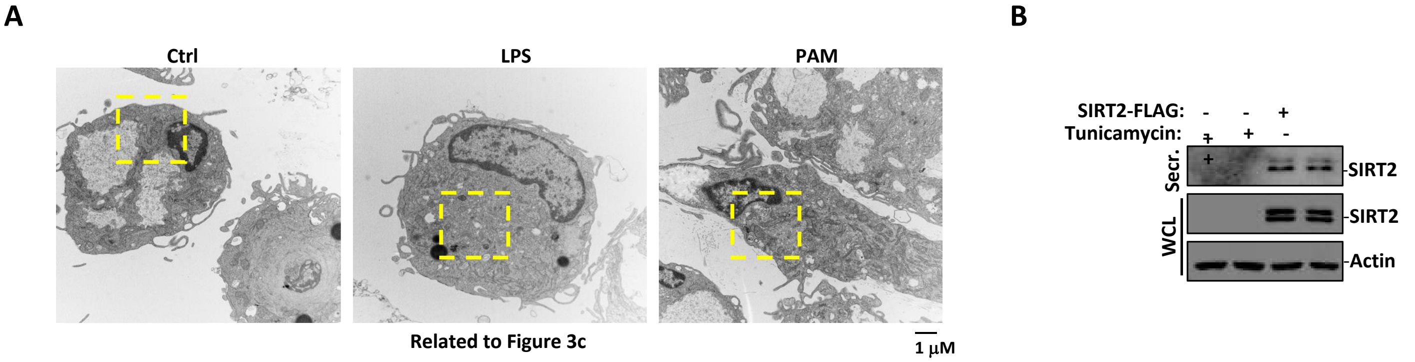

Figure S3. The autophagic machinery is utilized for SIRT2 secretion in Macrophages, related to Figure 3. (A) Representative TEM images of mouse peritoneal macrophages treated with LPS or PAM for 24 hrs under lower magnification in related to Figure 3c. Dashed rectangles depict the regions shown in Figure 3c. **(B)** HEK293T cells were transfected with SIRT2-Flag and treated with Tunicamycin (5 μg/ml). SIRT2 secreted into the medium were enriched by M2 beads for Western blot analysis with Flag antibody.

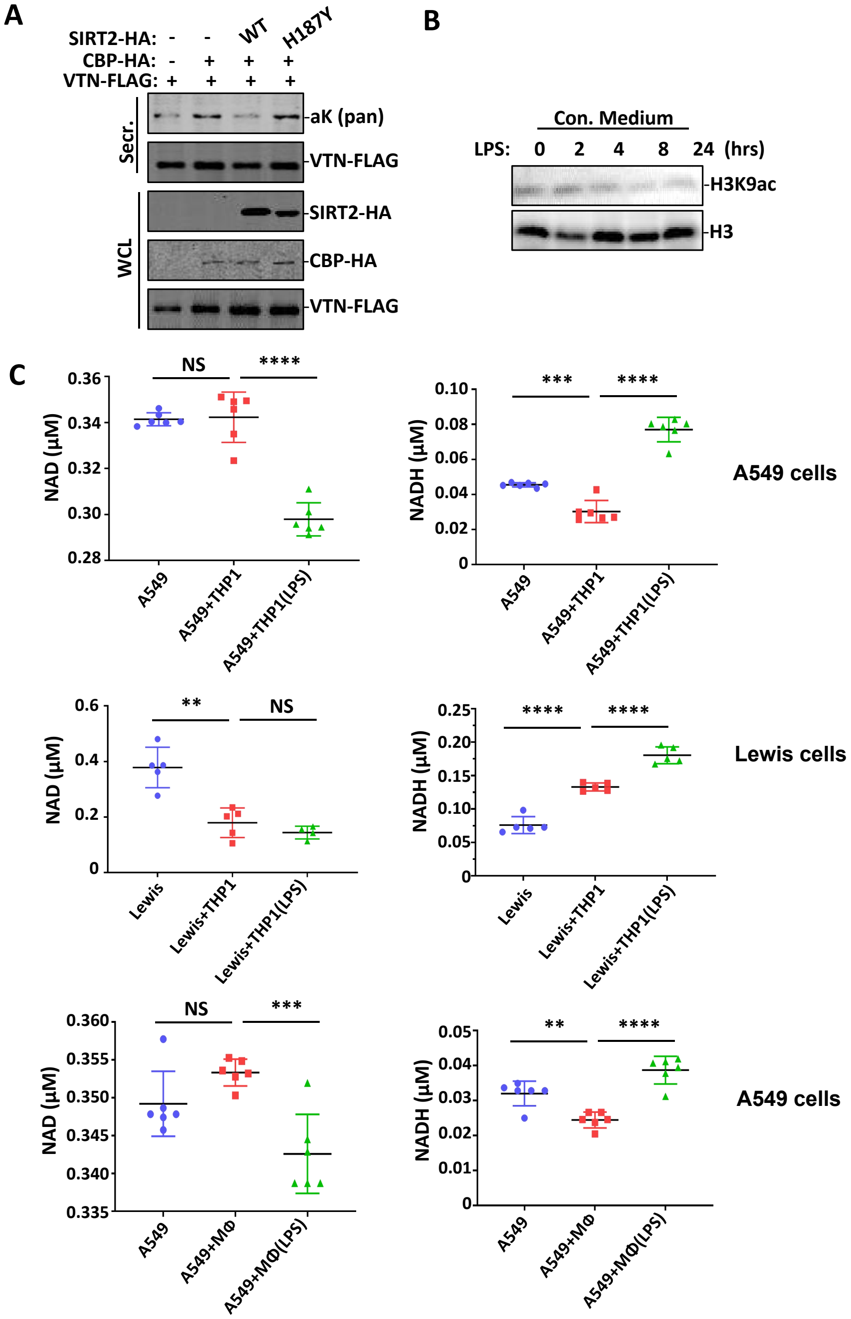

Figure S4. SIRT2 deacetylates other extracellular membrane proteins, related to Figure 6. (A) Wild type or H187Y mutated SIRT2 was transfected with CBP and VTN as indicated. Secreted VTN was was enriched with anti-FLAG M2 agarose beads from culture medium and subjected to Western blot analysis for acetylation analysis using antibodies as indicated. **(B)** THP1 cells were treated with LPS for different times as indicated. Conditional medium of these THP1 cell culture was collected and incubated with nuclear fractions obtained from J2 macrophages. H3K9ac and H3 were analyzed in Western blot. **(C)** Intracellular NAD^+ and NADH levels were measured. A549 cells or Lewis lung cancer cells were either cultured alone or cultured together with LPS pretreated (2 µg/ml, 12 hrs) macrophages (THP-1 cells or normal macrophages) for 24 hrs. Cells were then harvested for NAD^+ and NADH extraction and then measured using a NAD/NADH Quantitation Kit. Values were the mean ± SEM. ***p<0.01 ****p<0.001 *****p<0.0001 and NS no significance.

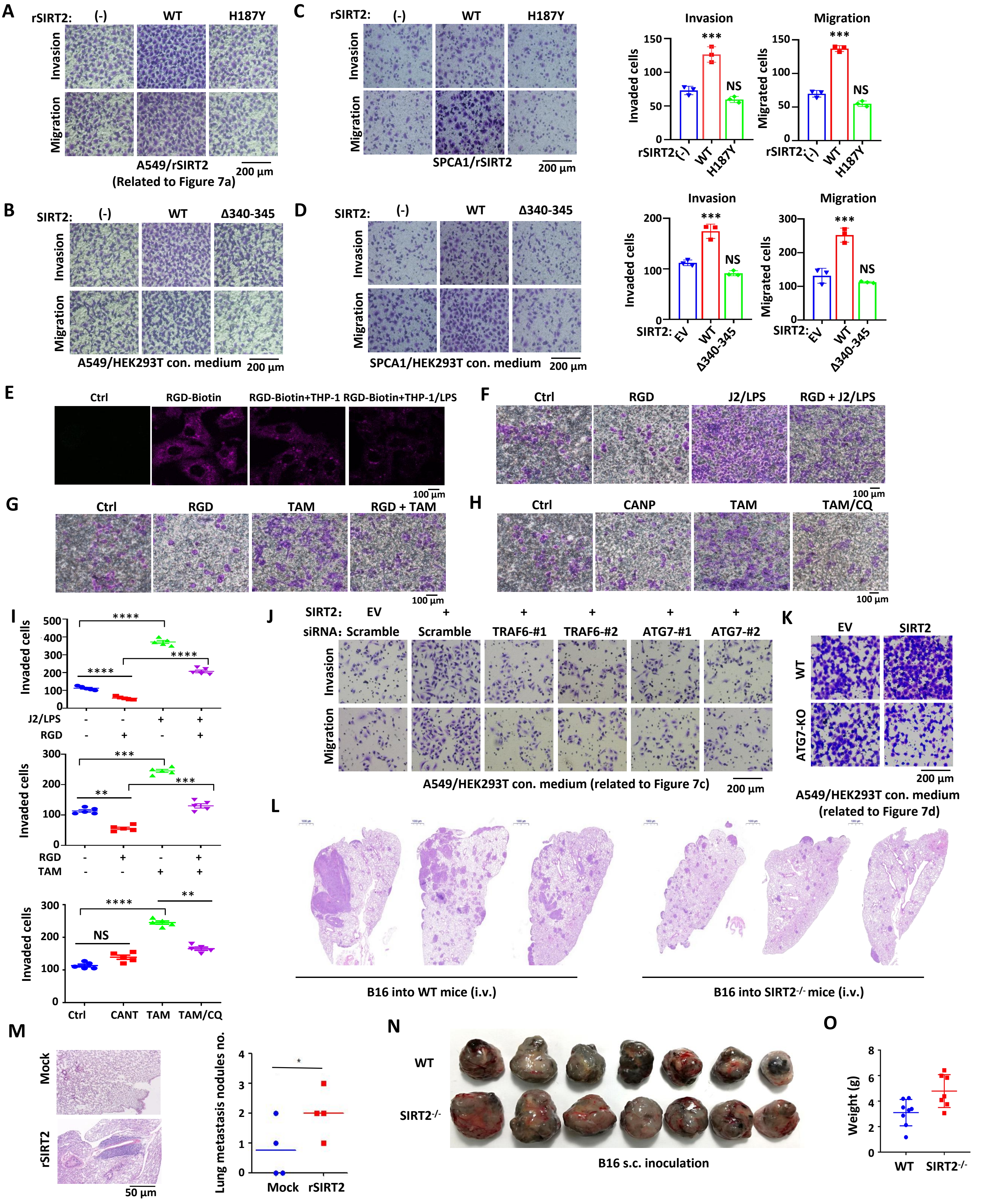

Figure S5. Secreted SIRT2 promotes lung cancer metastasis, related to Figure 7.

Figure S5. Secreted SIRT2 promotes lung cancer metastasis, related to Figure 7. (A-B) A549 cells were stained with crystal violet as described in Figure 7A-B. **(C)** Transwell assay of invasion and migration of SPCA1 cells using culture medium supplemented with purified recombinant SIRT2, wild type and H187Y as indicated (upper panels). Migrated (lower right) or invaded cells (lower left) were stained with crystal violet, counted and plotted in the lower panels. Data are shown as mean $\pm$ SEM. ***, $p<0.001$; NS indicates no significant difference between mock (-) and treatment groups. **(D)** SPCA1 cell migration and invasion towards culture medium from either SIRT2 WT-transfected or the SIRT2 Δ340-345-transfected HEK293T cells were determined by Transwell assay. Data are shown as mean $\pm$ SEM. ***, $p<0.001$; NS indicates no significant difference between EV and the treatment groups. **(E)** A549 cells were treated with RGD peptide-Biotin. Avidin-Alexa Fluor™ 647 signals were visualized with confocal fluorescent microscope. **(F)** Lewis lung cancer cells ($2x10^5$) were cultured alone or cocultured with LPS (2μg/ml, 12hrs)-activated J2 macrophages ($1x10^5$) followed by RGD peptide treatment. Twenty-four hours afterwards, Lewis lung cancer cells left in Boyden chamber were stained with crystal violet and photographed. **(G)** Similar to (F), Lewis lung cancer cells were cultured alone or cocultured with murine TAMs ($1x10^5$) followed by RGD peptide treatment for 24 hours. Lewis lung cancer cells left in Boyden chamber were stained with crystal violet and photographed. Columns represented mean $\pm$ SD of 3 experiments. **(H)** Lewis lung cancer cells ($2x10^5$) were cocultured with murine BMDMs or murine TAMs with or without 10μM Chloroquine (CQ) pretreatment for 2 hours. Twenty-four hours afterwards, Lewis lung cancer cells left in Boyden chamber were stained with crystal violet and photographed. Columns represented mean $\pm$ SD of 3 experiments. **(I)** Quantitative analysis of cell migration assay in (F-H). **(J-K)** Representative images of crystal violet-stained A549 cells as described in Figure 7C-D. **(L)** B16 cells were injected into wild type or $SIRT2^{-/-}$ mice through tail vein injection. Sixteen days post injection, the lung tissues were embedded in paraffin blocks and subjected to H&E staining. The whole slide imaging was performed using a DMetrix digital slide scanning instrument. **(M)** Lewis lung cancer cells ($5x10^6$) were injected subcutaneously alone or along with purified rSIRT2 proteins (140 μg) into the flank of male C57BL/6 mice for 6 weeks. The lung tissues were embedded in paraffin blocks and subjected to H&E staining. Quantification of lung microscopic nodules in the lungs of each group. Statistical analysis was performed using Student's t-test. *, $p<0.05$. **(N,O)** B16 cells were subcutaneously injected into the flanks of wild type or $SIRT2^{-/-}$ mice (N). Sixteen days post injection, mice were sacrificed and tumors were weighed (O).

A

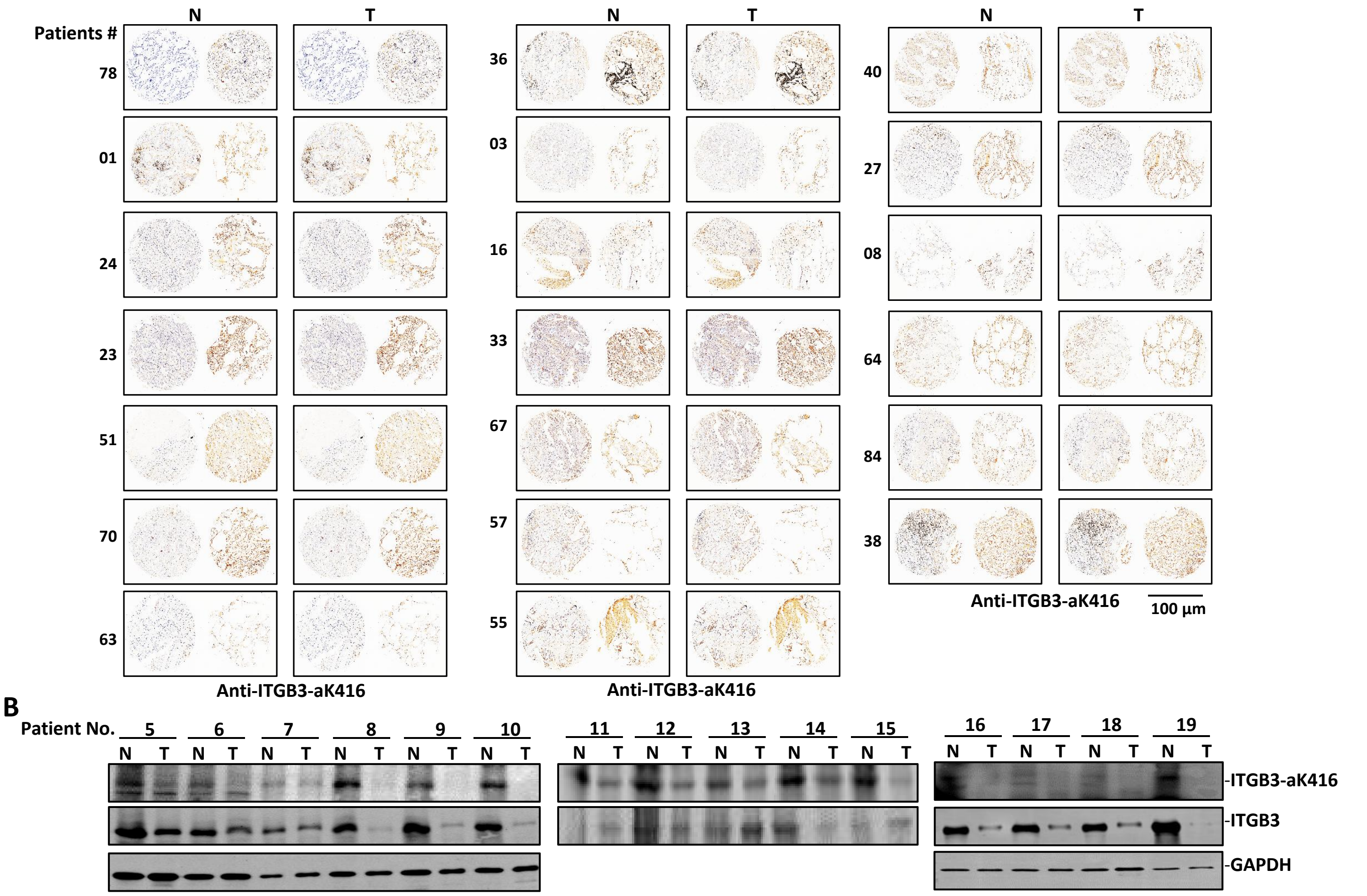

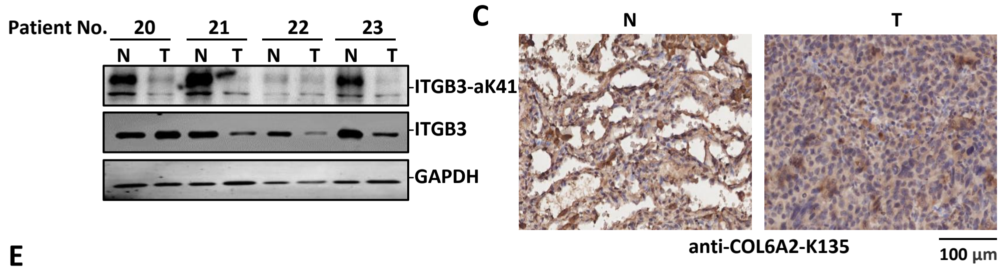

D

Patients characteristics	N(%)
Total number of patients	79
Gender<br>Male<br>Female	 40 (50.6%)<br>39 (49.4%)
Age (years)	46 (31-79)
EGFR amplification	39 (49.4%)
Histology<br>Adenocarcinoma<br>Squamous cell<br>Small cell	 79<br>0<br>0
Survival (months)	75.76 (3-125)
Clinical stage (TNM)<br>Ⅰ<br>Ⅱ<br>Ⅲ<br>Ⅳ	 24 (30.4%)<br>21 (26.6%)<br>11 (13.9%)<br>23 (29.1%)
Perineural invasion	0
Intravascular tumor thrombus	21 (26.6%)

E

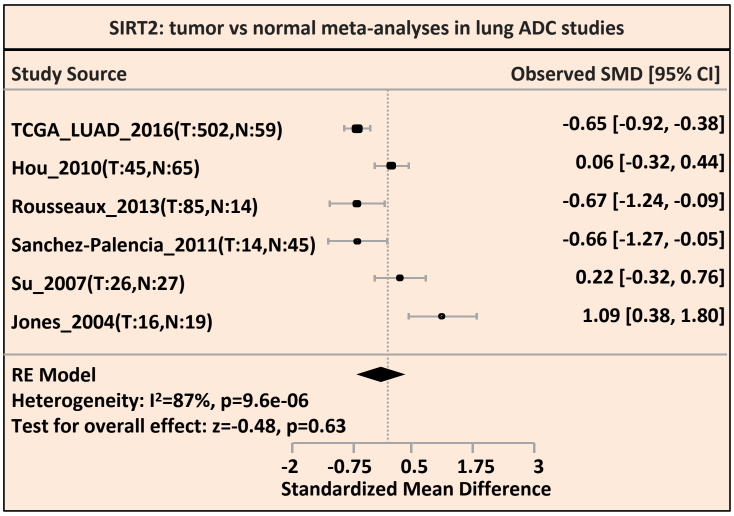

Figure S6. SIRT2 secretion and ITGB3 deacetylation correlate with poor prognosis in lung cancer, related to Figure 8.

Figure S6. SIRT2 secretion and ITGB3 deacetylation correlate with poor prognosis in lung cancer, related to Figure 8. (A) Tumor tissue samples and adjacent normal tissue samples (85 pairs) from LUAC patients were subjected to immunohistochemical analysis of ITGB3-K416 acetylation (tissue microarray). Among them, 20 pairs exhibited significant low ITGB3-K416 acetylation intensity in tumor tissue as compared with the adjacent normal tissue. **(B)** Lung tumor tissues and adjacent normal tissues were obtained from lung adenocarcinoma patients and subjected to Western blot analysis with antibodies against ITGB3-K416 acetylation and ITGB3. Representative immunoblots of samples from other individual patients (in relates to Figure 8f) were shown. **(C)** Immunohistochemical analysis of COL6A2-K135 acetylation in lung cancer tissue and adjacent normal tissue revealed no apparent difference in acetylation intensity. **(D)** Summary of clinical characteristics of 79 lung cancer patients. Total number of lung cancer patients was 85. **(E)** Forest plots for SIRT2 protein expression between normal individuals and patients with lung a Meta-analyses from 6 studies was performed on Lung Cancer Explorer (LCE) program with a web link (http://lce.biohpc.swmed.edu/).
